# Magnetically Navigated and PSMA/STEAP1 Dual-Targeted Nanoliposomes for NIR-II/MRI Imaging with Photothermal-Chemo-Immunotherapy for Prostate Cancer

**DOI:** 10.34133/research.1375

**Published:** 2026-08-14

**Authors:** Jian-Xuan Sun, Ye An, Si-Han Zhang, Jin-Zhou Xu, Maimaiti Abudureyimu, Jing-Yu Xu, Yi-Fan Huang, Yu-Xuan Yang, Mei-Cheng Liu, Shao-Gang Wang, Qi-Dong Xia

**Affiliations:** ^1^ Department and Institute of Urology, Tongji Hospital, Tongji Medical College, Huazhong University of Science and Technology, Wuhan, China.; ^2^ Department of Urology, The First People’s Hospital of Yunnan Province, Kunming University of Science and Technology Affiliated Hospital, Kunming, China.

## Abstract

Precision theranostics for castration-resistant prostate cancer remain a major challenge because of tumor heterogeneity, drug toxicity, and resistance to conventional therapies. Here, we report a multifunctional theranostic nanoplatform that integrates multimodal imaging guidance, dual-targeted delivery, photothermal therapy, and immune activation for the precision treatment of prostate cancer. By encapsulating aggregation-induced emission luminogens and magnetic nanoparticles, this system achieves superior second near-infrared fluorescence and magnetic resonance imaging fusion for deep-tissue visualization. The dual-targeting strategy, which integrates magnetic navigation with simultaneous engagement of six-transmembrane epithelial antigen of the prostate 1 and prostate specific membrane antigen (PSMA), effectively addresses tumor heterogeneity by enabling externally guided and biologically specific delivery to both PSMA^+^ and PSMA^−^ prostate cancer cells. Upon targeted delivery, the synergistic combination of photothermal therapy and chemotherapy achieves potent tumor growth inhibition, activates CD8^+^ T cells and macrophages to promote robust antitumor immune responses, and elicits durable immunological memory to prevent tumor recurrence. In vitro and in vivo studies demonstrate superior tumor targeting specificity, enhanced imaging contrast, and minimal off-target toxicity. This study provides novel insights into the construction of a multifunctional theranostic nanoplatform in precision oncology, with promising translational potential for clinical management of castration-resistant prostate cancer.

## Introduction

Prostate cancer (PCa) ranks as the most commonly diagnosed malignancy in men in more than half of the countries worldwide [1]. For patients with metastatic PCa, the 5-year survival rate is only ~30%, and curative management primarily relies on radical prostatectomy and androgen deprivation therapy (ADT) [2]. However, positive surgical margins and disease progression frequently lead to recurrence and metastasis, with 10% to 20% of advanced cases evolving into ADT-resistant castration-resistant prostate cancer (CRPC) within 5 years [3,4]. The resistance of CRPC to ADT represents a major clinical challenge in treatment. Although chemotherapy, poly (ADP-ribose) polymerase (PARP) inhibitors, and ^177^Lu-based radionuclide therapy have yielded incremental benefits for patients with CRPC, their long-term efficacy remains limited by pronounced molecular heterogeneity, therapeutic resistance, and unavoidable systemic toxicities [5,6]. Addressing these challenges requires not only more effective therapeutic agents but also strategies that enable real-time treatment monitoring, selective tumor accumulation, and multimechanism attack on resistant cells. Therefore, integrating precise imaging, tumor-specific targeting, and synergistic therapy represents a key direction for advancing CRPC treatment.

In recent years, nanomedicine has opened new avenues for the precision theranostics of cancer [7,8]. Owing to its ability to achieve spatiotemporally controlled therapeutic effects in response to external stimuli, photothermal therapy has emerged as an important modality in cancer treatment [9]. Additionally, aggregation-induced emission (AIE) materials can be engineered as near-infrared II (NIR-II) fluorescent probes. Leveraging the intrinsic advantages of NIR-II light, including deep-tissue penetration and minimal background interference, these probes have already found extensive applications in real-time, high-precision in vivo imaging of hepatocellular carcinoma tumors [10]. Intraoperative fluorescence-guided imaging enables clear delineation of tumor margins and real-time monitoring of the drug enrichment process at tumor sites, thereby providing intuitive evidence for surgical navigation and disease assessment [11]. On the therapeutic front, AIE materials can serve as core building blocks for constructing multifunctional theranostic platforms that integrate imaging guidance with photothermal therapy, chemotherapy, and other therapeutic modalities [12]. Meanwhile, magnetic nanomaterials such as Fe₃O₄ have been extensively integrated into multifunctional nanoplatforms for magnetic targeting, magnetic resonance imaging (MRI), and magnetic hyperthermia-based cancer treatment [13].

Precise tumor targeting can markedly enhance drug safety and remains a long-standing challenge to be addressed with regard to drug delivery. Engineered nanodelivery systems represent the primary approach to achieving specific tumor targeting [14]. In the clinical management of PCa, the prostate specific membrane antigen (PSMA)-targeted strategy represents the most commonly adopted approach [15,16]. However, targeting a single surface biomarker confers only limited efficacy in PCa and thus falls short of the rigorous demands of precision therapy [17]. PSMA expression displays pronounced multilevel heterogeneity: Merely 32.2% of CRPC cases exhibit high PSMA expression, a proportion that plummets to a mere 13.6% in neuroendocrine PCa. Notably, the emergence of treatment-induced neuroendocrine PCa is frequently accompanied by diminished PSMA expression, and similarly low PSMA expression is observed in liver metastases [18,19]. Therefore, the targeted treatment of PSMA-negative PCa has become an urgent challenge that demands immediate attention [20]. Six-transmembrane epithelial antigen of the prostate 1 (STEAP1) is also a PCa-specific transmembrane protein, and it is ubiquitously expressed in metastatic PCa [21]. In the targeted diagnosis and therapy of PCa, the dual-targeting strategy combining PSMA and STEAP1 enables broader coverage of tumor cell populations and reduces the risk of tumor escape. Distinct from molecular targeting strategies that rely on specific tumor surface biomarkers, physical targeting exploits the intrinsic magnetism of magnetic nanoparticles (MNPs) to achieve externally controlled drug delivery [22]. By integrating magnetic particle-mediated targeting with MRI, such magnetically guided nanoplatforms enable simultaneous noninvasive tumor imaging and localized therapeutic delivery [23]. Importantly, this magnetically controlled strategy operates independently of the expression levels of tumor cell surface biomarkers, thereby overcoming limitations imposed by tumor heterogeneity. As a result, physical targeting substantially enhances both the precision and the broad applicability of tumor-targeted diagnosis and therapy [24].

In the context of pharmacological therapy for PCa, the integration of chemotherapy with emerging therapeutic modalities has shown great promise in overcoming treatment resistance. Iron (II,III) oxide (Fe₃O₄) nanoparticles (NPs) are rich in both ferrous ions (Fe^2+^) and ferric ions (Fe^3+^), which participate in intracellular iron metabolism and facilitate the production of reactive oxygen species (ROS) via Fenton reaction, thereby enhancing oxidative stress-mediated tumor cell death [25]. Doxorubicin (DOX) induces tumor cell death through intercalative DNA binding and topoisomerase II inhibition, triggering DNA injury and cell apoptosis [26]. Furthermore, photothermal therapy has emerged as a minimally invasive treatment modality that converts NIR light into localized hyperthermia, leading to direct tumor ablation while simultaneously triggering immunogenic signals within the tumor microenvironment [27]. Moreover, photothermal therapy-induced thermal stress can trigger immunogenic cell death (ICD) and promote antitumor immune responses, including CD8^+^ T cell activation and macrophage polarization [28].

To the best of our knowledge, multifunctional nanoplatforms that combine NIR-II laser-induced photothermal therapy with integrated diagnosis and targeted therapy for PCa remain underexplored. Although multifunctional nanodelivery systems have been extensively investigated, few studies have adequately elucidated whether synergistic effects or mutual influences exist among the various components and functionalities. Herein, we constructed a multifunctional lipid NP platform that integrates precision imaging, targeted delivery, and synergistic therapy for PCa. Specifically, we developed AIE-MNPs/DOX@S/P-lip, a magnetically navigated, NIR-II/MRI dual-modal liposome encapsulating AIEgens for high-sensitivity tumor visualization. To overcome tumor heterogeneity, the system employs a dual-targeting strategy that combines the physical magnetic responsiveness of Fe₃O₄ NPs with the biological specificity of single-chain variable fragment (scFv) against PSMA and STEAP1, enabling precise delivery to both PSMA^+^ and PSMA^−^ cells. Furthermore, the Fe^3+^/DOX-coordinated payload exhibits strong tumor growth suppression, accompanied by activation of CD8^+^ T cells and macrophages for improved antitumor immunity and durable immunological memory.

## Results

### Synthesis and characterization of PoMVT, AIE-MNPs, and AIE-MNPs/DOX@S/P-lip

**Fig. 1.**
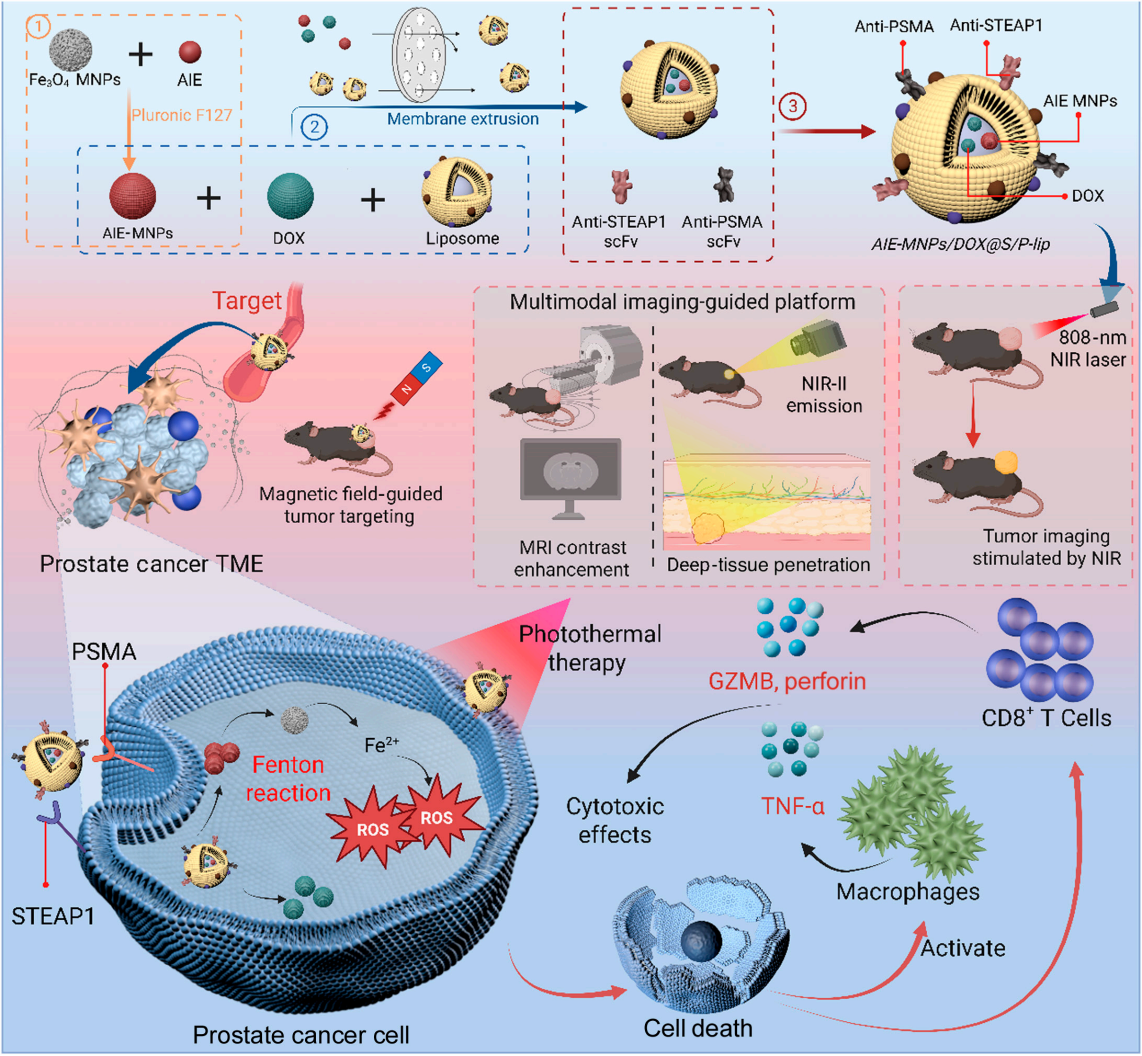
Schematic of this study.

**Fig. 2.**
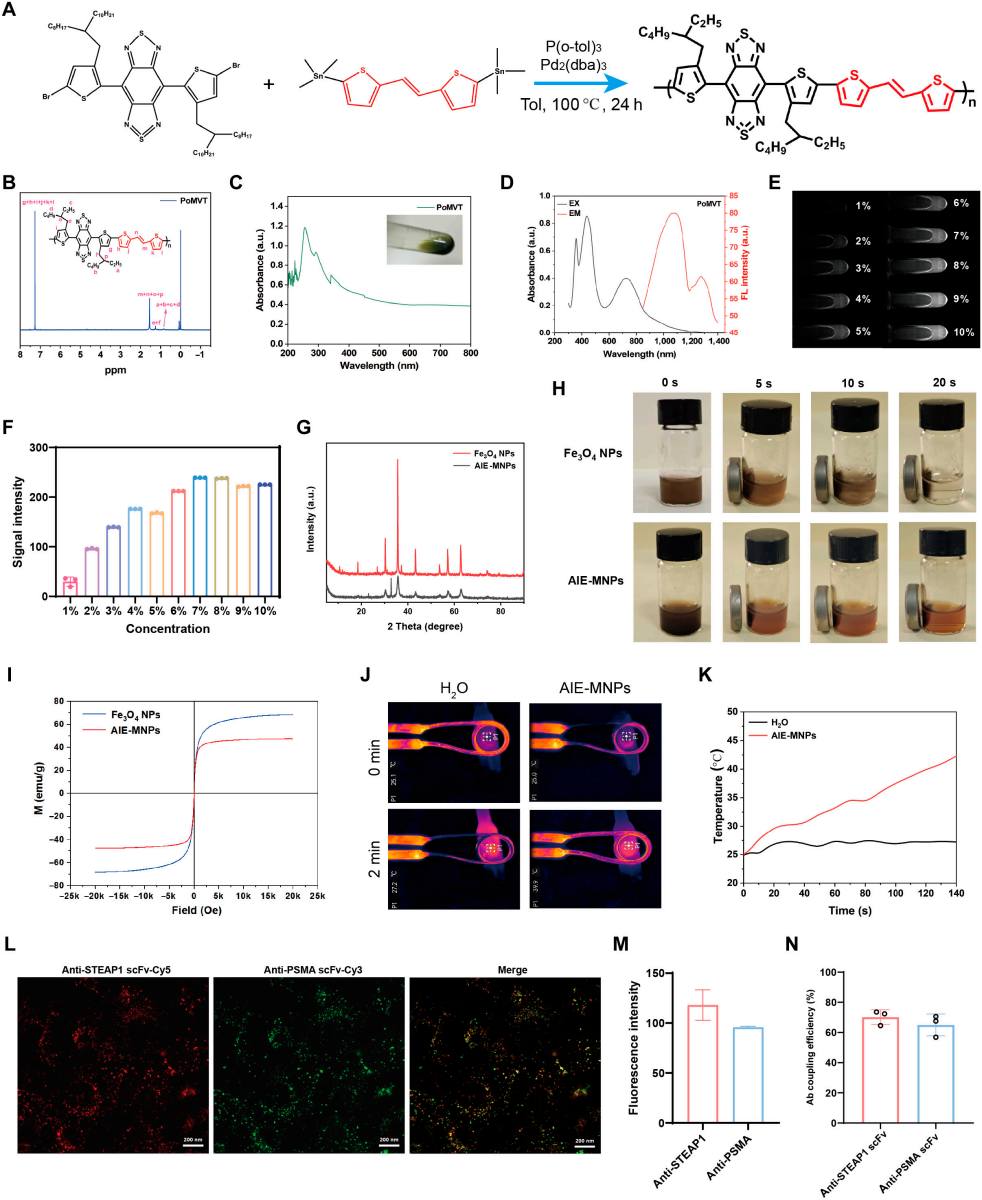
The synthesis and characterization of poly(octyl-modified benzobisthiadiazole-vinylene-bithiophene) (PoMVT) and aggregation-induced emission (AIE)-magnetic nanoparticles (MNPs). (A) Flow chart of the process of PoMVT synthesis. (B) The ^1^H nuclear magnetic resonance spectrum confirms the successful synthesis of PoMVT. (C) The ultraviolet–visible (UV–Vis) absorption spectrum shows the absorption band range and maximum absorption peak of PoMVT in the visible-near-infrared (NIR) region. (D) The fluorescence excitation/emission spectra detect the excitation and emission peaks of PoMVT. (E and F) The imaging performance (E) and signal intensity (F) of PoMVT at different concentrations (1% to 10%) in the NIR-IIa region are shown. (G) The x-ray diffraction clearly confirms the successful preparation and crystallinity of the Fe_3_O_4_ magnetic core. (H and I) The magnetic responsiveness (H) and magnetic regression curve (I) of Fe_3_O_4_ nanoparticles (NPs) and AIE-MNPs under an external magnetic field are presented. (J and K) Magnetothermal performance of AIE-MNPs under a high-frequency alternating magnetic field (450 kHz). (L and M) Super-resolution imaging via direct stochastic optical reconstruction microscopy verifies the successful modification and spatial distribution of scFvs (scale bars, 200 nm). (N) Conjugation efficiency of six-transmembrane epithelial antigen of the prostate 1 (STEAP1) and prostate specific membrane antigen (PSMA) scFv determined by UV spectrophotometry-based protein quantification.

The overall design and proposed therapeutic mechanism of this study are schematically illustrated in Fig. 1. As depicted in Fig. 2A, the AIE photosensitizer poly(octyl-modified benzobisthiadiazole-vinylene-bithiophene) (PoMVT) copolymer was successfully synthesized. ^1^H nuclear magnetic resonance spectroscopy fully confirmed the synthesis of PoMVT (Fig. 2B). The ultraviolet–visible (UV–Vis) absorption spectrum showed that PoMVT exhibited broad absorption characteristics from UV to NIR, with the strongest absorption peak at 250 nm, followed by a gradually decaying broad absorption band in the 300- to 700-nm region. This typical D-A type conjugated polymer absorption pattern reflected the effective intramolecular charge transfer effect between the benzobisthiadiazole acceptor and the thiophene donor (Fig. 2C). The fluorescence excitation/emission spectra revealed a large Stokes shift, with excitation peaks at 450 and 650 nm, and red-shifted emission peaks at 1,080 nm in the NIR-IIa window, minimizing self-absorption and enhancing imaging signal-to-noise ratios (Fig. 2D). We assessed the AIE properties of PoMVT by preparing solutions across a 1% to 10% concentration range and analyzing their fluorescence in the NIR-IIa window (Fig. 2E and F). Fluorescence intensity initially rose with increasing concentration, peaking at 7% to 8%, before declining. This optimal concentration delivered the strongest fluorescence signal and satisfied therapeutic drug loading needs. For imaging, excitation at 808 nm paired with an 1,100-nm long-pass filter optimized the signal-to-noise ratio.

We subsequently synthesized Fe₃O₄ NPs and AIE-modified MNPs (AIE-MNPs). The x-ray diffraction patterns revealed typical Fe₃O₄ diffraction peaks, with sharper peaks for Fe₃O₄ NPs indicating higher crystallinity, while AIE-MNPs showed reduced crystallinity due to AIE modification (Fig. 2G). Magnetic responsiveness tests revealed that both NPs responded to external magnetic fields, with Fe₃O₄ NPs adsorbing rapidly within 5 s, while AIE-MNPs showed slower adsorption and better redispersibility postmagnetic field removal, attributed to increased hydrophilicity and stability from the AIE-modified layer. Magnetic regression curve indicated both materials were superparamagnetic with low coercivity (Fig. 2H and I). Under a high-frequency alternating magnetic field (450 kHz), the AIE-MNP suspension achieved a temperature rise from 25 to 39.9 °C within 2 min, whereas the control group (pure water) showed only negligible temperature fluctuation (Δ*T* = 2.1 °C), fully demonstrating the exceptional sensitivity of AIE-MNPs in converting magnetic energy into thermal energy (Fig. 2J and K).

Subsequently, we prepared AIE-MNPs/DOX@lip by extrusion of lipid membranes, DOX, and AIE-MNPs and successfully modified the surface of AIE-MNPs/DOX@lip with anti-PSMA scFv and anti-STEAP1 scFv antibodies to obtain AIE-MNPs/DOX@S/P-lip. dSTORM super-resolution imaging confirmed the successful modification of anti-PSMA scFvs and anti-STEAP1 scFvs on the surface of the liposomes (Fig. 2L). Fluorescence intensity quantification showed the high efficiency and controllability of the antibody conjugation reaction (Fig. 2M). UV spectrophotometry-based protein quantification revealed conjugation efficiencies of anti-STEAP1 scFv and anti-PSMA scFv on the liposomal surface of 70.17 ± 4.86% and 64.99 ± 7.22%, respectively. Both antibody fragments were stably decorated on the liposome surface with high loading capacity (Fig. 2N).

**Fig. 3.**
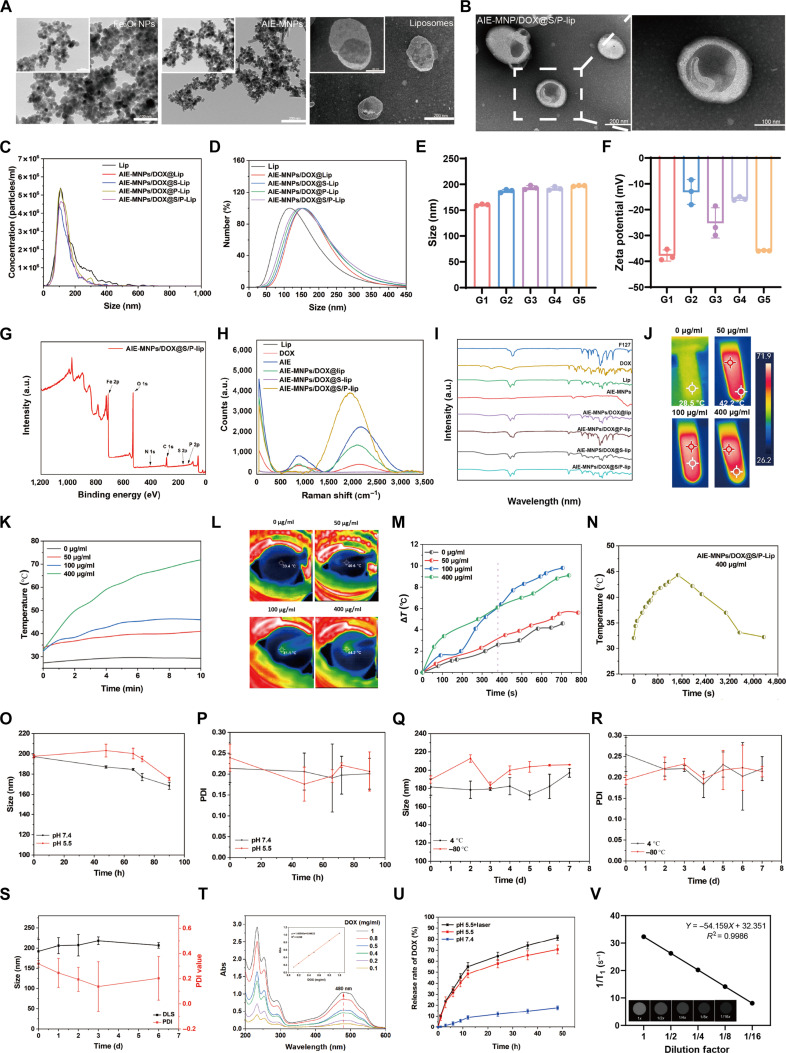
The characterization of AIE-MNPs/DOX@S/P-lip. (A and B) Transmission electron microscopy images of liposomes, Fe^3^O^4^ nanoparticles (NPs), AIE-MNPs, and AIE-MNPs/DOX@S/P-lip (scale bars, 200 nm). (C to E) The size distributions of G1 to G5 detected by nanoparticle tracking analysis (NTA) (C) and dynamic light scattering (DLS) (D and E). (F) Zeta potentials of G1 to G5 (G1: liposomes; G2: AIE-MNPs/DOX@lip; G3: AIE-MNPs/DOX@S-lip; G4: AIE-MNPs/DOX@P-lip; G5: AIE-MNPs/DOX@S/P-lip). (G) Depth-profiling x-ray photoelectron spectroscopy elucidates the chemical structure and interfacial interactions within AIE-MNPs/DOX@S/P-lip. (H) Raman spectroscopy fingerprints reveal molecular vibrations of each nanoassembly. (I) Fourier-transform infrared spectroscopy maps the chemical bonding and intermolecular interactions across the nanoplatform. (J and K) Photothermal conversion of AIE-MNPs/DOX@S/P-lip at varied concentrations. (L and M) Magnetothermal conversion of AIE-MNPs/DOX@S/P-lip at varied concentrations. (N) Cooling curves post heating. (O and P) pH stability of AIE-MNPs/DOX@S/P-lip: size (O) and polydispersity index (PDI) (P) of AIE-MNPs/DOX@S/P-lip at pH 7.4 or 5.5 over time. (Q and R) Thermal stability of AIE-MNPs/DOX@S/P-lip: size (Q) and PDI (R) after storage at 4 or −80 °C over time. (S) Stability of AIE-MNPs/DOX@S/P-lip NPs in serum measured by DLS. (T) DOX calibration curve and concentration-dependent absorbance. (U) DOX release profiles of AIE-MNPs/DOX@S/P-lip under different conditions. (V) T_1_-weighted relaxation rates and magnetic resonance images of AIE-MNPs/DOX@S/P-lip at different concentrations.

The structures of the individual components (Fig. 3A) and the final product (Fig. 3B) were observed via transmission electron microscopy (TEM). The initial Fe₃O₄ NPs exhibited a near-spherical morphology with an average particle size of 0.01 to 0.02 μm, displaying typical characteristics of superparamagnetic nanomaterials. After AIE functionalization, a distinct organic layer was observed on the surface of the NPs. The blank liposomes displayed a perfect unilamellar vesicle structure with a membrane thickness of approximately 5 to 7 nm, consistent with the theoretical thickness of a phospholipid bilayer. The final AIE-MNPs/DOX@S/P-lip exhibited a typical “raisin-bun” structure, with multiple MNP clusters effectively encapsulated within a single liposome. Negative staining clearly delineated the complete contour of the lipid bilayer membrane, and the overall particle size was controlled within the range of 150 to 180 nm. This size is conducive to tumor site enrichment via the enhanced permeability and retention effect while effectively avoiding rapid clearance by the reticuloendothelial system. Energy-dispersive x-ray spectroscopy mapping provided spatial distribution information of the individual components of the NPs (Fig. S1). Nanoparticle tracking analysis (Fig. 3C) and dynamic light scattering (DLS) (Fig. 3D) indicated that the particle size gradually increased with the encapsulation of AIE MNPs and DOX, as well as the modification of the 2 scFvs. The final average particle size was 197.17 nm, which fell within the optimal window for the enhanced permeability and retention effect (Fig. 3E). The zeta potential indicated that the final product had a stable potential of approximately −35.92 mV, indicating a highly stable structure (Fig. 3F). X-ray diffraction patterns confirmed the successful preparation and crystalline integrity of the Fe₃O₄ magnetic core (Fig. 3G). Raman spectroscopy (Fig. 3H), Fourier-transform infrared spectroscopy (Fig. 3I), and x-ray photoelectron spectroscopy (Fig. S2 and Table S1) demonstrated the successful integration of AIE materials, liposomes, chemotherapeutic drugs, and surface antibodies, with each component retaining its chemical integrity to form a synergistic functional system. Collectively, these results confirmed the successful synthesis of AIE-MNPs/DOX@S/P-lip.

### The photothermal and magnetothermal effects, stability, and DOX release profile of AIE-MNPs/DOX@S/P-lip

Then, the photothermal and magnetothermal effects, stability, and DOX release profile of AIE-MNPs/DOX@S/P-lip were systematically investigated. Photothermal studies revealed concentration-dependent heating, with AIE-MNPs/DOX@S/P-lip (400 μg/ml) achieving a maximum temperature rise of 43.4° C after 10 min of 808-nm irradiation. This confirmed the material’s excellent photothermal conversion capability (Fig. 3J and K). The photothermal conversion mechanism aligns with the classic Newton’s cooling law, achieving a photothermal conversion efficiency (η) of 67.4% (Fig. S3A and B). The thermal time constant τ_s_ = 1.76 min demonstrates the material has moderate thermal conductivity, enabling rapid heating while maintaining sufficient thermal retention, which is beneficial for effective photothermal therapy. The cyclic stability experiment confirmed the material’s photothermal stability and reusability (Fig. S3C). For AIE-MNPs/DOX@S/P-lip, the magnetothermal effect was not pronounced at a concentration of 50 μg/ml but became evident at 100 μg/ml, with the temperature rising and then gradually decreasing after the magnetic field was removed (Fig. 3L to N). These results demonstrate that AIE-MNPs/DOX@S/P-lip possesses excellent photothermal effects and moderate magnetothermal effects.

The stability and storage conditions of AIE-MNPs/DOX@S/P-lip were also investigated. Under pH 7.4 conditions, the NPs displayed remarkable colloidal stability, with their size gradually decreasing from 197.17 to 168.53 nm within 96 h, and the polydispersity index (PDI) remaining between 0.20 and 0.25. This indicates the integrity of the liposome structure and uniform dispersion. Under simulated tumor microenvironment conditions (pH 5.5), the particle size remained relatively stable within the initial 48 h, then rapidly decreased to 175.30 nm, with slight fluctuations in PDI but remaining below 0.25. This suggests partial disruption of the liposome structure in acidic conditions (Fig. 3O and P). When stored at 4 °C, the particle size fluctuated slightly within the range of 170 to 200 nm over 7 d, with the PDI maintained at 0.20 to 0.25, demonstrating the rationality of the formulation design and the structural integrity of the lipid bilayer at low temperatures. In contrast, storage at −80 °C led to pronounced instability, with drastic particle size fluctuations and increased PDI variability (0.15 to 0.28). This indicates that refrigeration at 2 to 8 °C is the optimal storage condition (Fig. 3Q and R). During the 6-d serum incubation period, the hydrodynamic size of AIE-MNPs/DOX@S/P-lip NPs remained stably maintained within the 190- to 220-nm range, exhibiting only minor fluctuations without appreciable aggregation or abrupt increase (Fig. 3S).

The release profile of DOX from AIE-MNPs/DOX@S/P-lip was analyzed using UV–Vis spectroscopy. The absorption spectra of DOX solutions at different concentrations (0.1 to 1.0 mg/ml) were measured, and a concentration–absorbance standard curve was plotted (Fig. 3T). Screening of various lipid-to-drug ratios revealed that as the ratio increased from 10:1 to 40:1, the encapsulation efficiency steadily improved from 89.9 ± 1.8% to 95.2 ± 1.6%, whereas the drug loading capacity decreased from 8.2 ± 0.4% to 2.3 ± 0.1%. Taking these factors into comprehensive consideration, a lipid-to-drug ratio of 20:1 was selected as the optimal formulation for subsequent experiments (Fig. S3D). Under pH 7.4 conditions, only 17.70 ± 1.72% of DOX was slowly released within 48 h, showing excellent circulation stability. However, in a simulated lysosomal acidic environment (pH 5.5), the release rate significantly increased to 70.73 ± 3.96%. More importantly, 808-nm laser irradiation further enhanced the 48-h release rate to 81.42 ± 2.27% (Fig. 3U). These results confirm that AIE-MNPs/DOX@S/P-lip exhibits substantially increased drug release in tumor microenvironments and under NIR irradiation, providing a foundation for achieving tumor-specific, on-demand drug delivery.

Finally, T_1_-weighted MRI contrast enhancement was assessed by linear regression of relaxation rates versus nanocomposite dilution (Fig. 3V). MRI scan showed that the signal intensity of the weighted imaging gradually darkened with increasing dilution ratios of AIE-MNPs/DOX@S/P-lip. The linear fitting results demonstrated a strong linear correlation between the dilution ratios and T_1_-weighted relaxation rates. These results indicate the potential of AIE-MNPs/DOX@S/P-lip as an effective MRI contrast agent for theranostic applications.

### In vitro cellular uptake and cytotoxicity of AIE-MNPs/DOX@S/P-lip

We evaluated the in vitro uptake of AIE-MNPs/DOX@S/P-lip by various PCa cell lines at different time points using flow cytometry (Fig. S4A to C). After 3 h of incubation, the uptake efficiency of all 3 cell lines achieved 90%. Next, we assessed concentration- and time-dependent cytotoxicity against PCa cell lines via Cell Counting Kit-8 (CCK-8) assay (Fig. S5A to J). AIE-MNPs/DOX@S/P-lip exhibited dose-responsive and temporally enhanced antiproliferative effects, with 10 μg/ml inducing significant growth inhibition across all cell lines after 48 h. Critically, cytotoxic potency was substantially greater in cell lines with high PSMA and STEAP1 expression (22Rv1, C4-2, and LNCaP), suggesting receptor-mediated therapeutic enhancement.

To further explore whether the cytotoxicity was dependent on the expression levels of PSMA and STEAP1, we established PC-3 with high expression of PSMA and STEAP1 (PC-3-PSMA and PC-3-STEAP1) and evaluated the cytotoxicity after 48 h of incubation (Fig. S5K and L). Although the cytotoxicity against PC-3-PSMA and PC-3-STEAP1 did not significantly increase at high concentrations (>10 μg/ml), the elevated expression of PSMA and STEAP1 enhanced the cytotoxicity at low concentration. This could be attributed to the binding of AIE-MNPs/DOX@S/P-lip to PSMA and STEAP1 on the cell surface, which facilitated its intracellular uptake and increased its intracellular concentration.

Flow cytometry analysis also revealed that AIE-MNPs/DOX@S/P-lip at 10 μg/ml significantly induced apoptosis in PCa cells (Fig. S6A to C). Moreover, transwell assays (Fig. S7A) and colony formation assays (Fig. S7B) indicated that AIE-MNPs/DOX@S/P-lip could markedly suppress the growth and migration of PCa cells.

### In vivo antitumor efficacy and safety of AIE-MNPs/DOX@S/P-lip

After confirming the in vitro cytotoxicity of AIE-MNPs/DOX@S/P-lip, we further evaluated its antitumor efficacy and safety in vivo (Fig. S8A). Tumor-bearing nude mice received intravenous injections of AIE-MNPs/DOX@S/P-lip (2, 3, or 5 mg/kg) every 3 d for 3 cycles. The results indicated that 2 mg/kg AIE-MNPs/DOX@S/P-lip significantly suppressed the growth of subcutaneous 22Rv1 xenografts. However, increasing the dose to 3 or 5 mg/kg did not markedly enhance the antitumor efficacy (Fig. S8B to E). All treatment groups showed no significant body weight loss (Fig. S8F), and serum biochemical analysis revealed no treatment-related alterations in hepatic or renal function markers (Fig. S8G to J). Histopathological examination of major organs showed no evidence of toxicity across all dose groups (Fig. S9K). Additionally, we assessed the hemolytic activity of AIE-MNPs/DOX@S/P-lip at various concentrations in vitro (Fig. S9A and B) and further evaluated its safety in C57BL/6 mice (Fig. S9C to G). The hemolytic assay results indicated that even at 200 μg/ml, AIE-MNPs/DOX@S/P-lip induced negligible hemolysis comparable to phosphate-buffered saline (PBS), and demonstrated excellent biocompatibility in C57BL/6J mice at all tested concentrations. These findings establish potent antitumor efficacy with a favorable safety profile. Based on the saturation of therapeutic response above 2 mg/kg and the absence of added benefit at 5 mg/kg versus 3 mg/kg, we selected 3 mg/kg as the optimal dose for subsequent studies. Representative hematoxylin and eosin staining of major organs was conducted 1 month after consecutive AIE-MNPs/DOX@S/P-lip administration (Fig. S9I). No noticeable pathological lesions were detected in tested visceral tissues, which verified the favorable long-term biosafety of AIE-MNPs/DOX@S/P-lip in vivo.

### In vitro and in vivo targeting ability of AIE-MNPs/DOX@S/P-lip

**Fig. 4.**
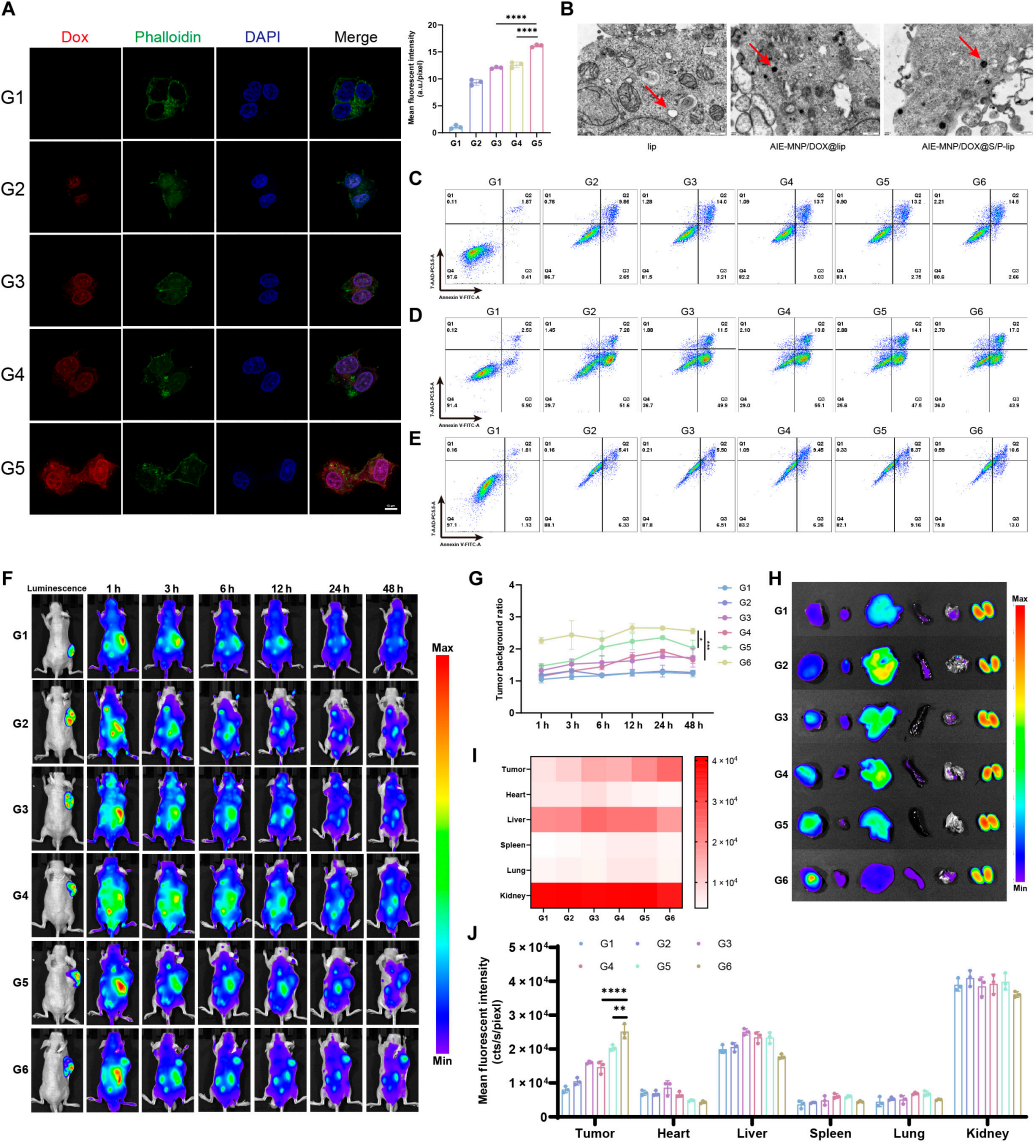
In vitro and in vivo targeting ability of AIE-MNPs/DOX@S/P-lip and transcriptomic sequencing analysis. (A) Immunofluorescence imaging of the uptake efficiency of different treatments by 22Rv1 at 3 h and average fluorescence intensity of doxorubicin in each group in immunofluorescence staining (scale bars, 10 μm; G1: liposomes; G2: AIE-MNPs/DOX@lip; G3: AIE-MNPs/DOX@S-lip; G4: AIE-MNPs/DOX@P-lip; G5: AIE-MNPs/DOX@S/P-lip). DAPI, 4′,6-diamidino-2-phenylindole. (B) Transmission electron microscopy analysis of cellular uptake of different treatments (scale bars, 500 nm). (C to E) Flow cytometry analysis of apoptosis induction in RM1 (C), 22Rv1 (D), and PC-3 (E) cells by different treatments (G1: liposomes; G2: DOX@lip; G3: AIE-MNPs/DOX@lip; G4: AIE-MNPs/DOX@S-lip; G5: AIE-MNPs/DOX@P-lip; G6: AIE-MNPs/DOX@S/P-lip). (F) Representative in vivo fluorescence images of tumor-bearing nude mice at different time points (1, 3, 6, 12, 24, and 48 h) after different treatment. Tumor location was identified by bioluminescence (G1: liposomes; G2: AIE-MNPs/DOX@lip; G3: AIE-MNPs/DOX@S-lip; G4: AIE-MNPs/DOX@P-lip; G5: AIE-MNPs/DOX@S/P-lip; G6: AIE-MNPs/DOX@S/P-lip + external magnetic field). (G) Tumor-to-background ratios at the indicated time points. (H to J) Ex vivo fluorescence imaging (H) and quantitative analysis (I and J) of major organs at 48 h.

We further explored the targeting ability of AIE-MNPs/DOX@S/P-lip both in vitro and in vivo. Dual-targeted AIE-MNPs/DOX@S/P-lip achieved significantly higher internalization in PSMA^+^/STEAP1^+^ 22Rv1 cells compared to all control formulations (Fig. 4A). TEM also confirmed effective cellular uptake (Fig. 4B). To functionally validate receptor-mediated delivery, we assessed cytotoxicity against PCa cell lines with varying PSMA/STEAP1 expression. In PSMA^−^/STEAP1^−^ RM1 cells, dual-targeted AIE-MNPs/DOX@S/P-lip exhibited no enhanced cytotoxicity relative to precursor formulations (Fig. 4C and Fig. S10A). Conversely, in PSMA^+^/STEAP1^+^ 22Rv1 cells, dual targeting significantly increased the proportion of late-apoptotic cells without affecting overall apoptosis rate (Fig. 4D and Fig. S10B and C). In PC-3 cells with low PSMA/STEAP1 expression, only modest enhancement in apoptosis was observed (Fig. 4E and Fig. S10D). Collectively, these results demonstrate potent and selective targeting of PCa cells in a PSMA- and STEAP1-dependent manner.

We next evaluated in vivo tumor targeting using a 22Rv1 xenograft model in nude mice (Fig. 4F to J). Live imaging showed prominent accumulation in tumor tissues, peaking at 12 to 24 h postinjection, with concurrent sequestration in liver and kidneys. Compared to all control groups, AIE-MNPs/DOX@S/P-lip demonstrated significantly greater accumulation in tumor tissues. Dual-targeted nanocomposites showed superior tumor accumulation compared to all control formulations. To leverage magnetic responsiveness, we applied an external magnetic field to the tumor site postinjection. Although the magnetic field did not augment peak tumor accumulation, it markedly accelerated early tumor homing (within 3 h) and prolonged intratumoral retention beyond 24 h. Comparable magnetically enhanced targeting dynamics were observed in immunocompetent C57BL/6 mice bearing RM1-STEAP1-PSMA tumors (Fig. S11). In summary, AIE-MNPs/DOX@S/P-lip demonstrates robust receptor-mediated tumor targeting in vivo, and magnetic field augmentation further enhances both the kinetics and duration of intratumoral accumulation. Consistent with the in vivo tumor enrichment results, plasma pharmacokinetic analysis revealed prolonged systemic circulation of AIE-MNPs/DOX@S/P-lip; the formulation displayed gradual plasma concentration decay over 48 h, which facilitated sustained blood supply and continuous tumor accumulation.

### In vitro investigation of the photothermal and magnetothermal effects of AIE-MNPs/DOX@S/P-lip

After confirming the targeting ability and direct cytotoxic mechanisms of AIE-MNPs/DOX@S/P-lip both in vitro and in vivo, we further explored its photothermal and magnetothermal effects in vitro. Results showed that after 5 min of irradiation with 808-nm NIR light, the temperature of culture wells containing AIE-MNPs/DOX@S/P-lip rose to 43 °C, while wells with PBS showed negligible temperature change (Fig. S12A). However, even after 10 min of exposure to an alternating magnetic field, culture dishes with AIE-MNPs/DOX@S/P-lip exhibited no significant temperature increase compared to the PBS group (Fig. S12B).

Flow cytometric analysis of apoptosis and CCK-8 revealed that neither NIR irradiation nor the application of an alternating magnetic field had a cytotoxic effect on PCa cells in the PBS group. Notably, while 808-nm NIR irradiation enhanced the cytotoxicity of AIE-MNPs/DOX@S/P-lip against PCa cells, the application of magnetic field did not. Moreover, the magnetic field failed to synergize with NIR irradiation to augment cytotoxicity (Fig. S12C to F). These findings indicated that at the chosen drug concentration, AIE-MNPs/DOX@S/P-lip demonstrated effective photothermal therapy against PCa cells but showed less pronounced magnetothermal effects. Consequently, subsequent in vivo experiments focused primarily on evaluating its photothermal therapeutic potential.

Given that recent studies have shown that photothermal therapy can improve treatment efficacy by elevating intracellular ROS production in addition to directly killing cells through heat, we investigated whether photothermal therapy could further boost ROS generation induced by AIE-MNPs/DOX@S/P-lip. Results confirmed that AIE-MNPs/DOX@S/P-lip alone increased intracellular ROS levels, and 808-nm NIR irradiation further elevated ROS production, inducing oxidative stress and subsequent cell apoptosis (Fig. S12G to I).

### Transcriptomic sequencing unveils multimodal antitumor mechanisms of AIE-MNPs/DOX@S/P-lip

**Fig. 5.**
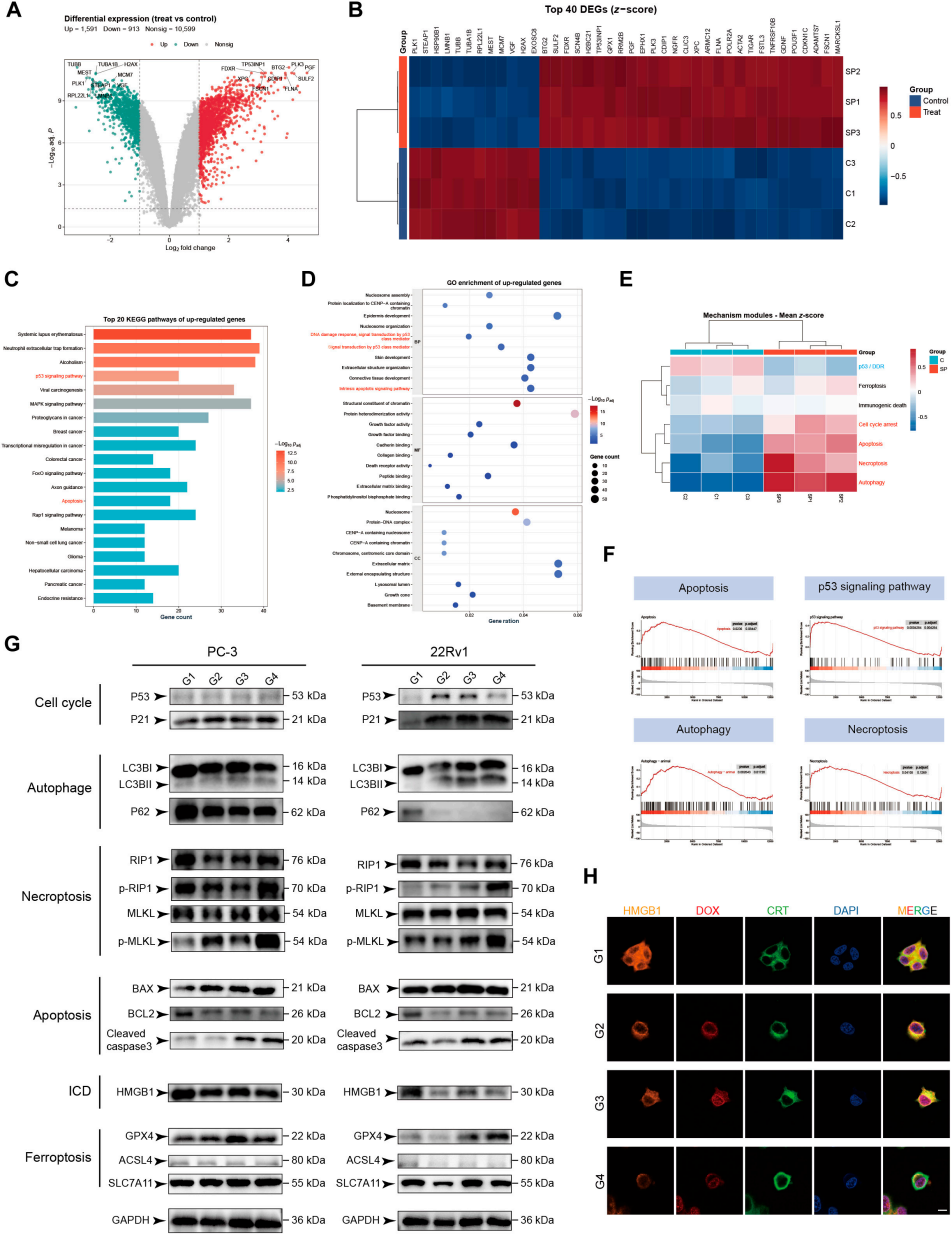
Transcriptomic profiling and experimental validation of the therapeutic mechanisms of AIE-MNPs/DOX@S/P-lip in 22Rv1 cells. (A) Volcano plot displaying differentially expressed genes (DEGs) in AIE-MNPs/DOX@S/P-lip-treated versus untreated control 22Rv1 cells. (B) Heatmap of the top 40 DEGs, showing distinct transcriptional segregation between control and AIE-MNPs/DOX@S/P-lip-treated groups. (C) Bar plot of the top 20 enriched Kyoto Encyclopedia of Genes and Genomes (KEGG) pathways for up-regulated DEGs, with color gradients representing enrichment scores. (D) Bubble plots for Gene Ontology (GO) functional enrichment of up-regulated DEGs. (E) Heatmap illustrating the averaged *z*-scores of core biological effects identified via Gene Set Enrichment Analysis (GSEA) enrichment analysis following AIE-MNPs/DOX@S/P-lip treatment. (F) GSEA enrichment plots of 4 core biological pathways, including apoptosis, p53 signaling pathway, autophagy, and necroptosis, in AIE-MNPs/DOX@S/P-lip-treated 22Rv1 cells. (G) Western blot of key functional proteins associated with cell cycle arrest, autophagy, necroptosis, apoptosis, immunogenic cell death (ICD), and ferroptosis in AIE-MNPs/DOX@S/P-lip-treated PC-3 and 22Rv1 cells. Glyceraldehyde phosphate dehydrogenase (GAPDH) was used as an internal loading control. (H) Representative immunofluorescence images of high mobility group box 1 (HMGB1) (orange), DOX (red), calreticulin (CRT) (green), nuclear 4′,6-diamidino-2-phenylindole (DAPI) staining (blue), and merged images in AIE-MNPs/DOX@S/P-lip-treated 22Rv1 cells (G1: phosphate-buffered saline; G2: AIE-MNPs/DOX@lip; G3: AIE-MNPs/DOX@S/P-lip; G4: AIE-MNPs/DOX@S/P-lip + laser).

To dissect the molecular mechanism underlying the cytotoxicity of the multimodal therapy, transcriptomic profiling of treated versus control PCa cells was first performed. We screened the top 40 differentially expressed genes (Fig. 5A and B). Combined with Gene Ontology (GO), top 20 Kyoto Encyclopedia of Genes and Genomes (KEGG) and Gene Set Enrichment Analysis (GSEA) analyses of differentially expressed genes, we verified the activation of p53 signaling, apoptosis, necroptosis, autophagy and cell cycle arrest; notably, GSEA further validated prominent enrichment of the p53 pathway (*P* = 0.00428) and autophagy signature (*P* = 0.01728) upon treatment (Fig. 5C to F). These pathways were validated at the protein level by Western blotting in 2 human PCa lines, PC-3 and 22Rv1. At the molecular level, treatment induced evident cell cycle arrest, accompanied by increased expression of p53 and p21. Enhanced autophagic activity was also observed, as reflected by the conversion of LC3B-I to LC3B-II and the down-regulation of p62. Moreover, elevated p-RIP1 and p-MLKL indicated the activation of cellular necroptosis. For apoptosis, the expression of proapoptotic proteins was increased while antiapoptotic protein levels were decreased, along with the up-regulation of cleaved caspase-3. In terms of ferroptosis, treated cells showed a compensatory up-regulation of GPX4, while other ferroptosis-related proteins exhibited no obvious changes (Fig. 5G).

To confirm ICD, 2 canonical hallmarks including surface exposure of calreticulin (CRT) and nuclear-to-extracellular release of high mobility group box 1 (HMGB1) were visualized by confocal immunofluorescence (Fig. 5H). In control G1 (PBS) cells, HMGB1 (orange) was distributed throughout the nucleus and cytoplasm, with minimal membrane-localized CRT (green) and no intracellular DOX fluorescence. After drug administration, red DOX autofluorescence was detected intracellularly in G2 to G4, confirming successful cellular drug uptake; G2 and G3 induced moderate increases in surface CRT and partial nuclear-to-cytoplasmic translocation of HMGB1, while these ICD-related changes were markedly augmented in the laser-combined G4 group, which exhibited the most robust ICD induction among all cohorts. CRT expression on cell surface rose sharply, whereas nuclear HMGB1 declined and the protein mainly relocated to cytoplasm, implying extracellular leakage of HMGB1. The above ICD phenotypes were the most pronounced in the G4 group.

### In vivo investigation of the direct cytotoxicity and photothermal therapeutic efficacy of AIE-MNPs/DOX@S/P-lip

**Fig. 6.**
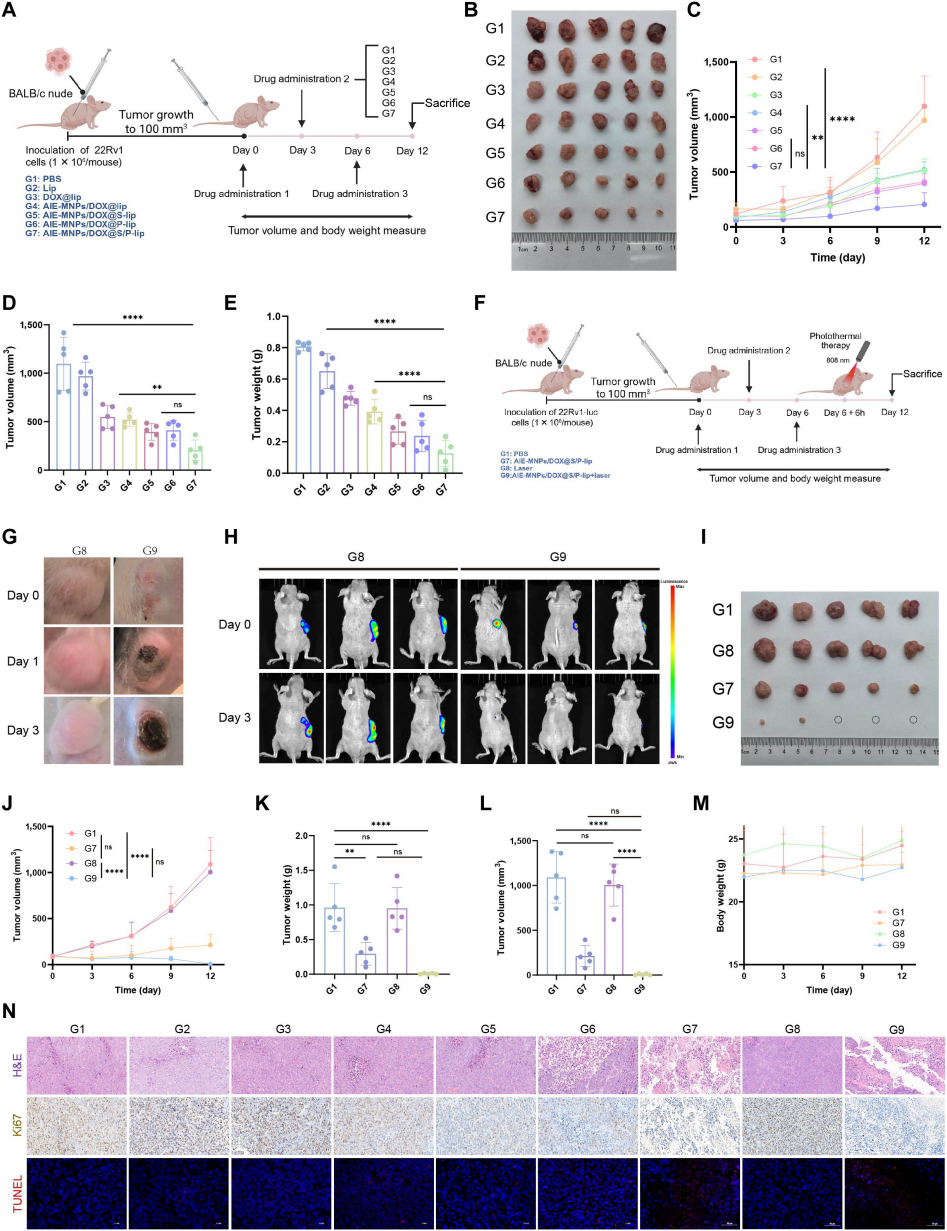
In vivo investigation of the direct cytotoxicity and photothermal therapeutic efficacy of AIE-MNPs/DOX@S/P-lip. (A) The animal experimental procedure. (B) Ex vivo images of tumors after mouse sacrifice (*n* = 5) (G1: phosphate-buffered saline; G2: liposomes; G3: DOX@lip; G4: AIE-MNPs/DOX@lip; G5: AIE-MNPs/DOX@S-lip; G6: AIE-MNPs/DOX@P-lip; G7: AIE-MNPs/DOX@S/P-lip). (C) The tumor growth curves (*n* = 5). (D and E) The comparison of tumor volume (D) and weight (E) after mouse sacrifice (*n* = 5). (F) The animal experimental procedure. (G and H) Photographs and bioluminescence images of the tumor region before and after photothermal therapy (G8: PBS + laser; G9: AIE-MNPs/DOX@S/P-lip + laser). (I) Ex vivo images of tumors after mouse sacrifice (*n* = 5) (G1: PBS; G7: AIE-MNPs/DOX@S/P-lip; G8: PBS + laser; G9: AIE-MNPs/DOX@S/P-lip + laser). (J) The tumor growth curves (*n* = 5). (K and L) The comparison of tumor volume (L) and weight (K) after mouse sacrifice (*n* = 5). (M) The body weight of mouse after drug administration (*n* = 5). (N) The hematoxylin and eosin (H&E) staining, terminal deoxynucleotidyl transferase dUTP nick end labeling (TUNEL) immunofluorescence staining and Ki67 immunohistochemical staining in tumor sections (scale bar, 50 μm). Data are presented as means ± SD. ns, no significance; *P < 0.05, **P < 0.01, ***P < 0.001, ****P < 0.0001.

After confirming the targeting ability, intrinsic cytotoxicity, and photothermal potential of AIE-MNPs/DOX@S/P-lip in vitro, we further evaluated its direct cytotoxicity (Fig. 6A) and photothermal therapeutic efficacy (Fig. 6F) in nude mice xenograft models. Results showed that compared to PBS, lip, DOX@lip, and AIE-MNPs/DOX@lip, AIE-MNPs/DOX@S/P-lip significantly inhibited tumor growth (Fig. 6B and C), reduced the final tumor volume and mass (Fig. 6D and E), and showed a declining trend in final tumor volume and mass compared to AIE-MNPs/DOX@S-lip and AIE-MNPs/DOX@P-lip. Additionally, all groups caused no significant reduction in mouse body weight (Fig. S13A), and no treatment-related alterations in liver or kidney function (Fig. S13B to E) or in the histology of major organs were observed. Compared to the laser-only group, the AIE-MNPs/DOX@S/P-lip group showed significant tumor necrosis 24 h postlaser irradiation (Fig. 6G). Bioluminescence imaging indicated that 72 h postirradiation, tumors in the AIE-MNPs/DOX@S/P-lip group nearly completely regressed, while tumors in the PBS group continued growing (Fig. 6H). Compared to the PBS, laser-only, and AIE-MNPs/DOX@S/P-lip groups, the AIE-MNPs/DOX@S/P-lip plus laser group demonstrated remarkable suppression of tumor cell growth (Fig. 6I and J), significantly reduced final tumor volume and mass (Fig. 6K and L), and achieved complete tumor regression in 60% of mice. Notably, photothermal therapy was very safe, causing no significant reduction in mouse body weight or morphological damage to major organs (Fig. 6M and Fig. S14). Hematoxylin and eosin, Ki67, and terminal deoxynucleotidyl transferase dUTP nick end labeling (TUNEL) staining collectively indicated the most potent antitumor effects in G7 and G9, characterized by severe tissue necrosis, markedly reduced proliferation, and extensive apoptosis, compared to G1 to G5 (Fig. 6N). Collectively, these findings indicated that compared to control groups, AIE-MNPs/DOX@S/P-lip exerted significant direct cytotoxicity and showed safety in vivo, while photothermal therapy further augmented its antitumor efficacy.

### In vivo investigation of the near-infrared fluorescence and MRI imaging ability of AIE-MNPs/DOX@S/P-lip

**Fig. 7.**
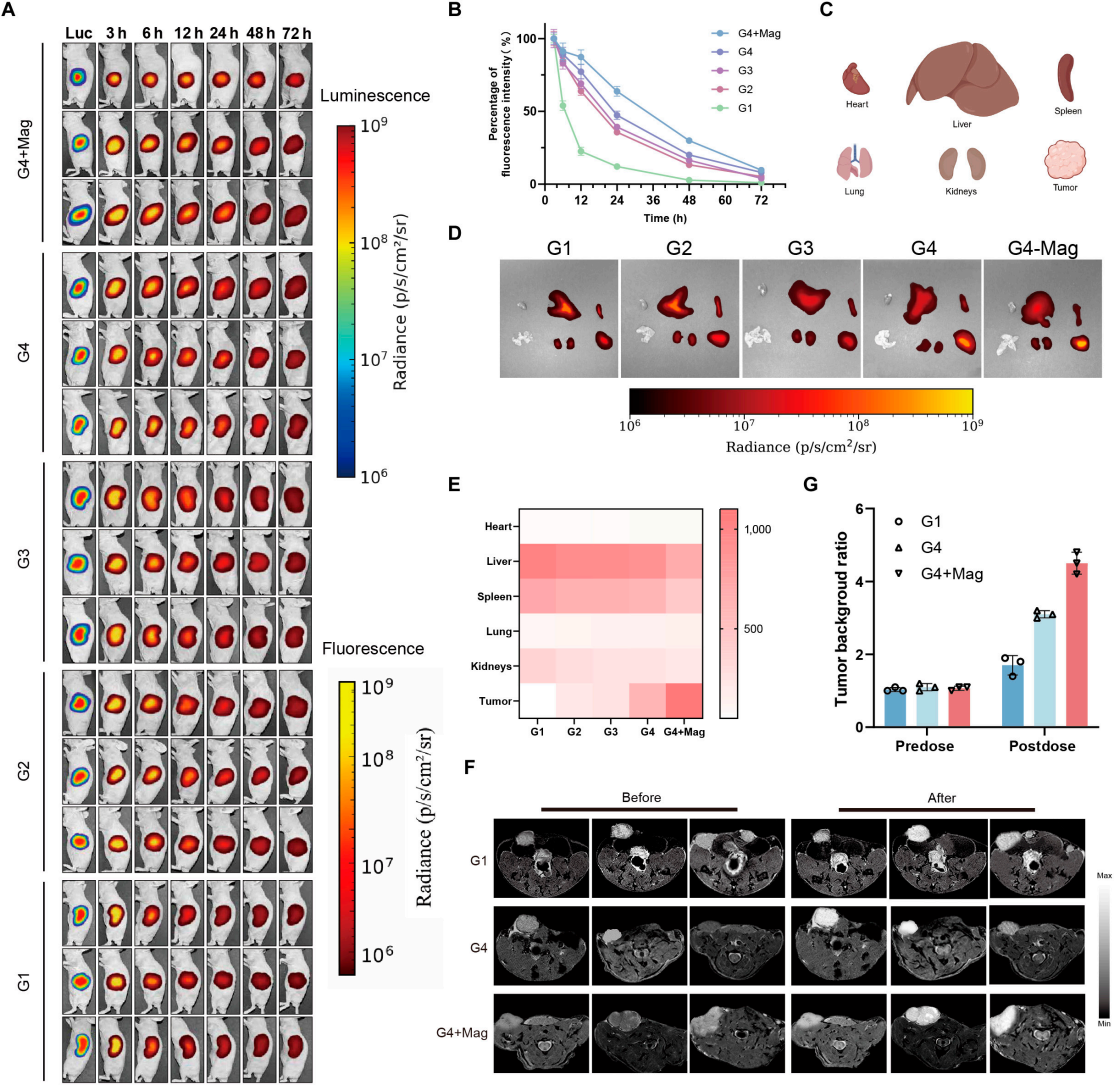
In vivo investigation of the near-infrared fluorescence and magnetic resonance imaging (MRI) ability of AIE-MNPs/DOX@S/P-lip. (A) Bioluminescence imaging of mouse tumors and near-infrared II (NIR-II) in vivo imaging of mice at different time points (3, 6, 12, 24, 48, and 72 h) (G1: phosphate-buffered saline; G2: AIE-MNPs/DOX@S-lip; G3: AIE-MNPs/DOX@P-lip; G4: AIE-MNPs/DOX@S/P-lip; G4 + Mag: AIE-MNPs/DOX@S/P-lip + external magnetic field). (B) Relative fluorescence intensity decay curve in the tumor region (*n* = 3). (C) Schematic diagram of organ locations. (D and E) NIR-II imaging of ex vivo organs at 72 h (D) and their relative fluorescence intensities (E). (F and G) MRI images of mouse before administration and 24 h postadministration (F) and the tumor-to-background signal ratio of MRI (G).

We further evaluated the near-infrared fluorescence and MRI imaging ability of AIE-MNPs/DOX@S/P-lip using a 22Rv1-luc xenograft model in nude mice. Before imaging, bioluminescence imaging was used to assess tumor growth (Fig. 7A). Mice were then randomly assigned to 4 groups and administered equal doses of PBS, AIE-MNPs/DOX@S-lip, AIE-MNPs/DOX@P-lip, or AIE-MNPs/DOX@S/P-lip via tail vein injection. Mice injected with AIE-MNPs/DOX@S/P-lip were further divided into 2 subgroups: one with an external magnetic field applied to the tumor site for 10 min postinjection and one without. NIR-II fluorescence imaging was performed at 3, 6, 12, 24, 48 and 72 h postinjection. Results indicated that fluorescence intensity at tumor sites peaked at 3 h and gradually declined thereafter. AIE-MNPs/DOX@S/P-lip showed precise tumor enrichment with the slowest decay rate among all groups. The application of an external magnetic field further slowed the fluorescence intensity decline at the tumor site (Fig. 7B). Postimaging at 72 h, mice were sacrificed, and fluorescence intensity in various organs was measured (Fig. 7C to E). Results showed that except for liver, spleen, and kidneys, AIE-MNPs/DOX@S/P-lip primarily accumulated in tumors, exhibiting significantly higher tumor fluorescence intensity than control groups. The external magnetic field group demonstrated even higher tumor fluorescence intensity, consistent with earlier findings.

For MRI imaging, T_1_-weighted images were acquired before and 24 h postinjection (Fig. 7F and G). Preinjection MRI signal intensity in the tumor region was comparable across all groups. However, 24 h postinjection, AIE-MNPs/DOX@S/P-lip plus external magnetic field group showed significantly higher MRI signal intensity in the tumor region compared to other groups, indicating effective MRI targeting of tumors under external magnetic field guidance. Overall, these results demonstrate that AIE-MNPs/DOX@S/P-lip possesses excellent magnetism and multimodal imaging capabilities.

### In vivo investigation of the ICD induced by AIE-MNPs/DOX@S/P-lip combined with photothermal therapy

**Fig. 8.**
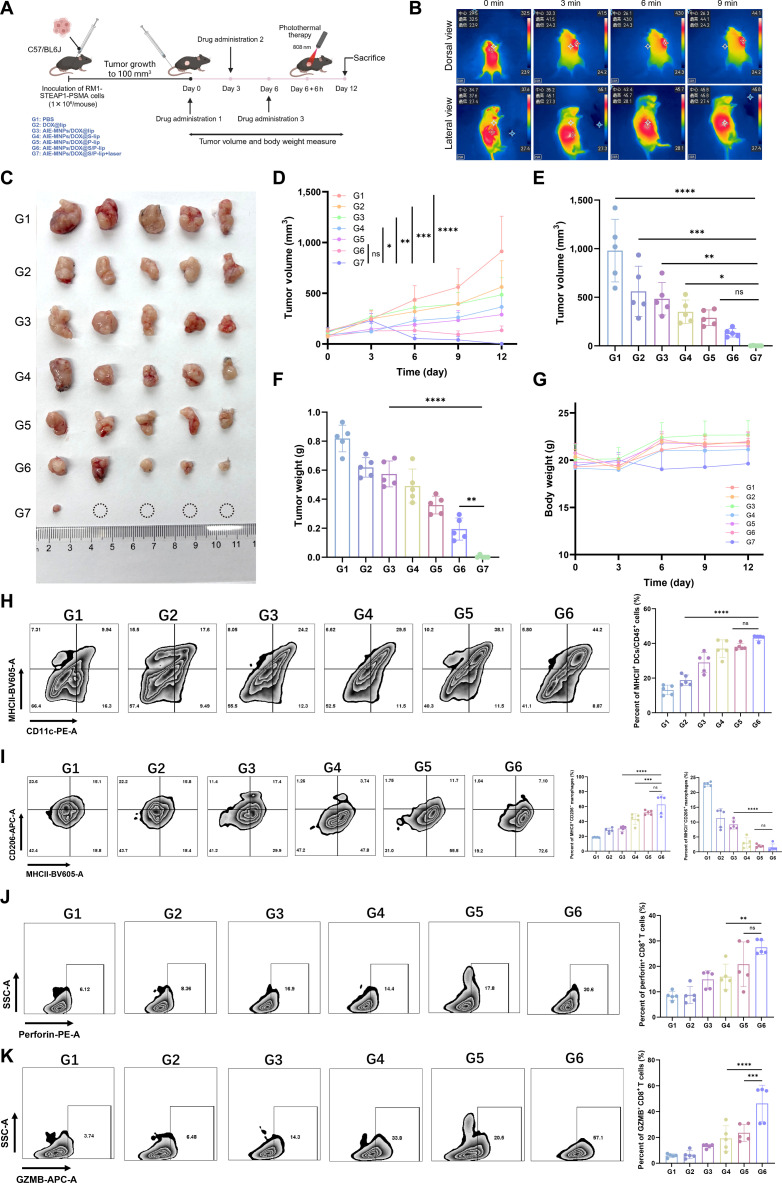
In vivo investigation of immunogenic cell death induced by AIE-MNPs/DOX@S/P-lip. (A) The animal experimental procedure. (B) Temperature change of mice before and after 808-nm near-infrared (NIR) laser irradiation for 10 min. (C) Ex vivo images of tumors after mouse sacrifice (*n* = 5) (G1: phosphate-buffered saline; G2: DOX@lip; G3: AIE-MNPs/DOX@lip; G4: AIE-MNPs/DOX@S-lip; G5: AIE-MNPs/DOX@P-lip; G6: AIE-MNPs/DOX@S/P-lip; G7: AIE-MNPs/DOX@S/P-lip + laser). (D) The tumor growth curves (*n* = 5). (E and F) The comparison of tumor volume (E) and weight (F) after mouse sacrifice (*n* = 5). (G) The body weight of mouse after drug administration (*n* = 5). (H to K) Representative flow cytometry images and quantitative analysis of the proportion of MHCII^+^DCs (H), MHCII^+^macrophages, CD206^+^macrophages (I), perforin^+^ (J), and granzyme B (GZMB^+^) (K) CD8^+^T cells. Data are presented as means ± SD. ns, no significance; *P < 0.05, **P < 0.01, ***P < 0.001, ****P < 0.0001.

In order to explore whether AIE-MNPs/DOX@S/P-lip combined with photothermal therapy could not only directly kill tumors but also activate antitumor immunity and induce ICD, we randomly divided the immunocompetent C57BL/6 mice bearing subcutaneous RM1-STEAP1-PSMA xenografts into 7 groups: PBS, DOX@lip, AIE-MNPs/DOX@lip, AIE-MNPs/DOX@S-lip, AIE-MNPs/DOX@P-lip, AIE-MNPs/DOX@S/P-lip, and AIE-MNPs/DOX@S/P-lip combined with laser irradiation (Fig. 8A). After laser irradiation, the temperature of the tumor area in the mice rose rapidly, exceeding 45 °C (Fig. 8B). Compared with other groups, AIE-MNPs/DOX@S/P-lip significantly reduced the tumor volume, and when combined with photothermal therapy, the tumor volume was further reduced, with tumors in 4 mice disappearing completely (Fig. 8C to F). Additionally, this treatment did not cause a significant reduction in body weight (Fig. 8G) or noticeable damage to the morphology of major organs (Fig. S15).

Flow cytometry revealed that AIE-MNPs/DOX@S/P-lip remarkably activated dendritic cells (DCs) (Fig. 8H and Fig. S17A and B), macrophages (Fig. 8I and Fig. S17C and D), and CD8^+^ T cells (Fig. 8J and K and Fig. S17E and F) within the tumor. It enhanced the antigen-presenting capacity of DCs, promoted the polarization of macrophages from the M2 to M1 phenotype, and increased the proportion of activated CD8^+^ T cells. This was accompanied by an elevated expression of antitumor molecules such as granzyme B, perforin, and interferon-γ (IFN-γ), leading to a substantial enhancement of systemic antitumor immunity.

**Fig. 9.**
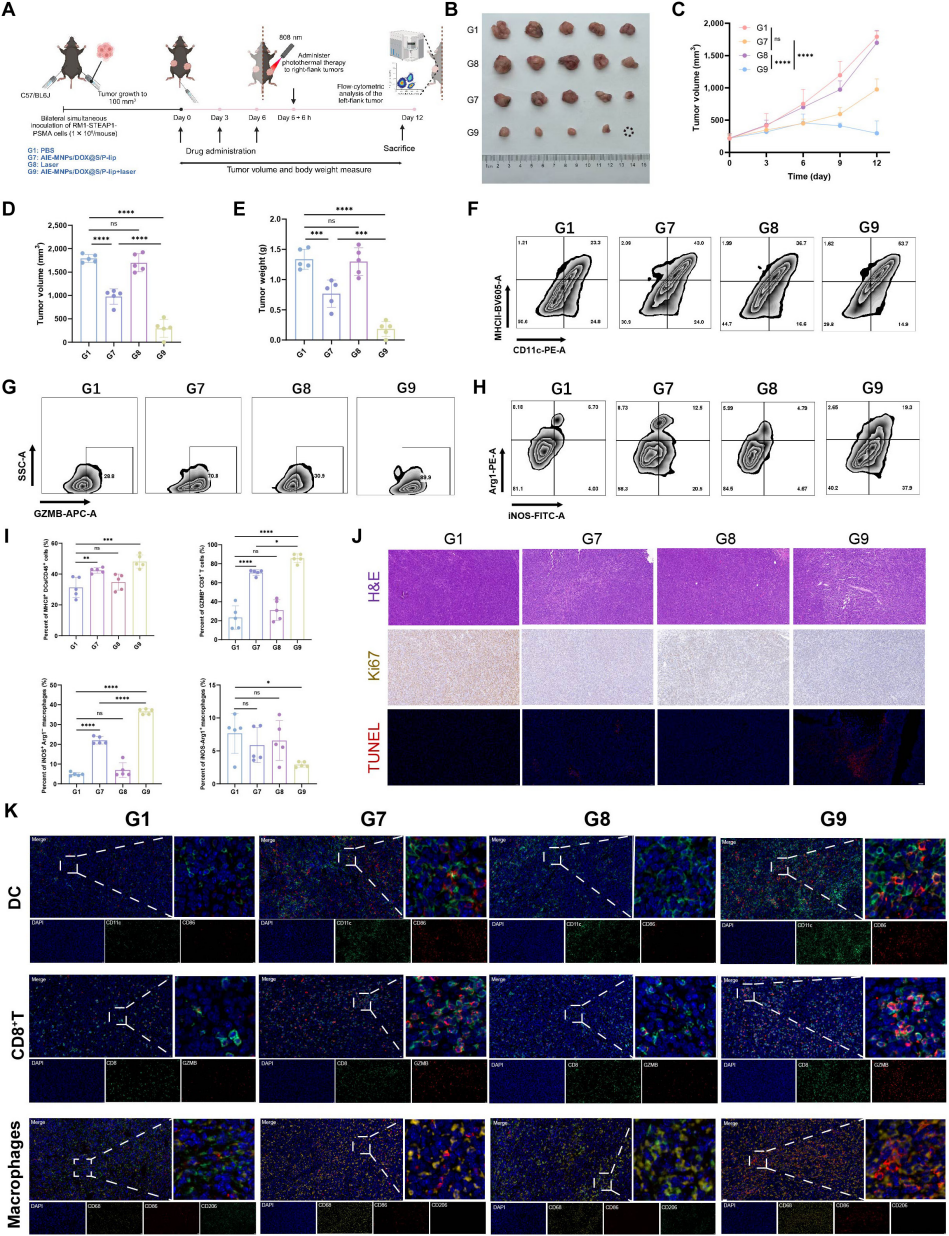
In vivo investigation of immunogenic cell death induced by AIE-MNPs/DOX@S/P-lip combined with photothermal therapy. (A) The animal experimental procedure. (B) Ex vivo images of left tumors after mouse sacrifice (*n* = 5). (C) The tumor growth curves of left tumors (*n* = 5). (D and E) The comparison of tumor volume (D) and weight (E) of left tumors after mouse sacrifice (*n* = 5). (F to I) Representative flow cytometry images of the proportion of MHCII^+^DCs (F), GZMB^+^CD8^+^T cells (G), iNOS^+^macrophages, Arg1^+^macrophages (H), and quantitative analysis (I). (J) The hematoxylin and eosin (H&E) staining, terminal deoxynucleotidyl transferase dUTP nick end labeling (TUNEL) immunofluorescence staining and Ki67 immunohistochemical staining in tumor sections (scale bar, 50 μm). (K) Immunofluorescence staining of different dendritic cell (DC) markers (CD86, CD11c), CD8^+^T cell markers (CD8 and granzyme B [GZMB]) and macrophages markers (CD68, CD86, and CD206) in tumor tissue sections (scale bar, 20 μm). Data are presented as means ± SD. ns, no significance; *P < 0.05, **P < 0.01, ***P < 0.001, ****P < 0.0001.

Since photothermal therapy led to complete tumor regression in most mice, making it impossible to effectively assess immune cell infiltration within the tumor, we inoculated subcutaneous xenografts on both sides of the mice. We applied photothermal therapy to the tumor on the right side and evaluated immune cell infiltration in the tumor on the left side to investigate whether photothermal therapy could further activate antitumor immunity (Fig. 9A). The results demonstrated that while AIE-MNPs/DOX@S/P-lip led to a significant reduction in tumor size on both sides, the combination with photothermal therapy resulted in complete regression of the right-side tumor without a noticeable reduction in body weight. Moreover, in mice subjected to photothermal therapy combined with AIE-MNPs/DOX@S/P-lip, the left-side tumor also exhibited reduced size after photothermal therapy (Fig. 9B to E and Fig. S18A to E). This was confirmed by Ki-67 immunohistochemical staining and TUNEL immunofluorescence, which showed that photothermal therapy suppressed tumor growth and enhanced apoptosis (Fig. 9J). Further flow cytometry analysis of immune cell infiltration in the left-side tumors showed remarkable activation of DCs (Fig. 9F and I and Fig. S19A and B), CD8^+^ T cells (Fig. 9G and H and Fig. S19C to E), and macrophages (Fig. 9H and I) in the combined therapy group. Immunofluorescence results also confirmed these findings (Fig. 9K). Collectively, these results highlight the ability of AIE-MNPs/DOX@S/P-lip combined with photothermal therapy to significantly activate antitumor immune responses and induce ICD of tumor cells.

### AIE-MNPs/DOX@S/P-lip combined with photothermal therapy elicits durable systemic CD8^+^ T cell memory and protects against tumor rechallenge

**Fig. 10.**
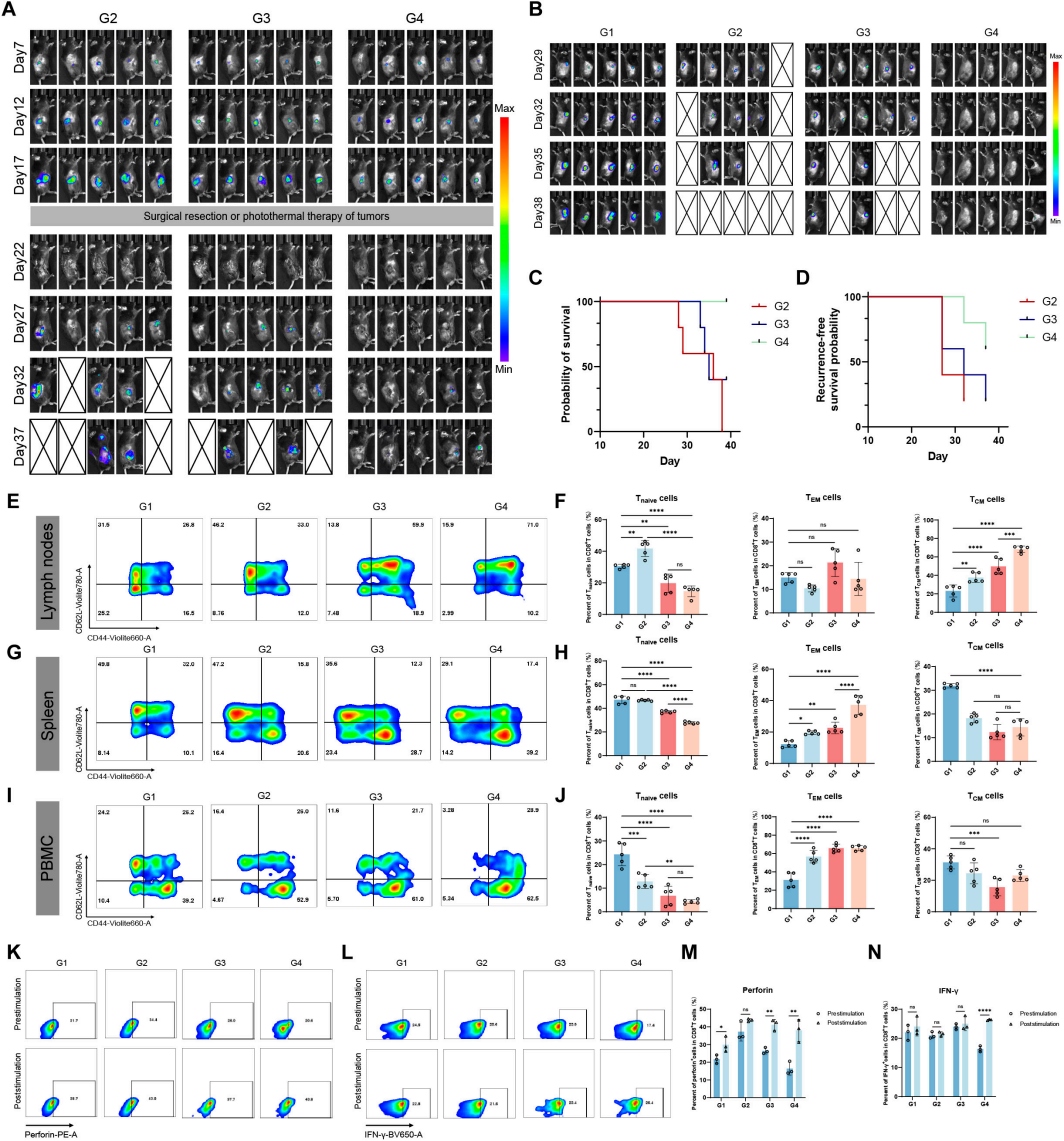
AIE-MNPs/DOX@S/P-lip combined with photothermal therapy elicits long-term immunological memory against tumor rechallenge. (A) Representative bioluminescence imaging of tumor recurrence in mice receiving different treatments (G2, G3, and G4) after primary tumor resection and photothermal therapy. Crosses indicate mice that were euthanized or had reached the humane end point. (B) Bioluminescence imaging of contralateral tumor rechallenge in the naive control and treatment groups (G1 to G4). Crosses indicate mice that were euthanized or had reached the humane end point. (C) Kaplan–Meier survival curves of mice in different groups (*n* = 5). (D) Recurrence-free survival probability of mice in different groups (*n* = 5). (E, G, and I) Representative flow cytometry plots showing the gating of naive T cells (T_naive_, CD44^−^CD62L^+^), central memory T cells (T_CM_, CD44^+^CD62L^+^), and effector memory T cells (T_EM_, CD44^+^CD62L^−^) in lymph nodes (E), spleen (G), and peripheral blood mononuclear cells (PBMCs; I) across different treatment groups (G1 to G4). (F, H, and J) Quantification of T_CM_, T_EM_, and T_naive_ cells in lymph nodes (F), spleen (H), and PBMCs (J) by flow cytometry. (K and L) Flow cytometry analysis showing the expression changes of perforin (K) and IFN-γ (L) in CD8^+^T cells from each group before and after ex vivo stimulation. (M and N) Quantification of the percentages of perforin^+^ (M) and IFN-γ^+^ (N) in CD8^+^ T cells across different groups (data are presented as means ± SD, *P < 0.05, **P < 0.01, ***P < 0.001, ****P < 0.0001. G1: blank; G2: phosphate-buffered saline; G3: AIE-MNPs/DOX@S/P-lip; G4: AIE-MNPs/DOX@S/P-lip + laser).

To determine whether the multimodal therapy conferred long-lived protective immunity, mice in the targeted chemo-photothermal group (G4, AIE-MNPs/DOX@S/P-lip + laser) that had achieved complete tumor clearance were selected; in the G2 and G3 groups, residual primary tumors were surgically resected. After a memory-consolidation interval, mice were rechallenged with RM1-STEAP1-PSMA-luc cells in the contralateral flank. In the G2 group, primary tumors recurred early and secondary tumors grew progressively, albeit slightly slower than those in the G1 group; both primary recurrence and rechallenge growth were markedly attenuated in the G3 and G4 groups (Fig. 10A and B). Suppression was most pronounced in mice that had received AIE-MNPs/DOX@S/P-lip combined with 808-nm laser irradiation (G4), with tumor recurrence detected in only 1 out of 5 animals at the contralateral site (Fig. 10B). Mice in the G2 group began to die on day 28, with 2 deaths observed by day 32 and complete mortality by day 38, whereas all mice in the G4 group remained alive on day 38 (Fig. 10C). Additionally, the G4 group exhibited a longer recurrence-free survival period compared to both the G2 and G3 groups (Fig. 10D).

To define the immunological basis of this protection, CD8^+^ T cells from lymph nodes, spleen, and peripheral blood were phenotyped by flow cytometry, resolving naive (T_naive_; CD44^−^CD62L^+^), central memory (T_CM_; CD44^+^CD62L^+^), and effector memory (T_EM_; CD44^+^CD62L^−^) subsets (Fig. S20A to D). The proportion of T_naive_ within the CD8^+^ compartment was lowest in the G4 group relative to the other groups. In the spleen and PBMC, T_EM_ cells were significantly increased in the G4 group, whereas no significant increase was observed in the lymph nodes (Fig. 10G to J). In contrast, T_CM_ cells were significantly elevated in the lymph nodes (Fig. 10E and F). Although we observed a decrease in T_CM_ cells in the spleen of the G3 and G4 groups, splenic enlargement was noted in both groups (Fig. S20E). We next interrogated the functional quality of this memory by restimulating splenocytes ex vivo with tumor antigen and quantifying effector-molecule production (Fig. 10K and L). The proportions of IFN-γ- and perforin-producing CD8^+^ T cells were markedly higher in the G4 group than in the G1 to G3 groups, confirming that the expanded memory cells remained antigen-responsive and retained cytotoxic effector capacity (Fig. 10M and N).

## Discussion

In the study, we successfully constructed a theranostic nanoplatform, denoted as AIE-MNPs/DOX@S/P-lip, which integrates NIR-II fluorescence imaging, targeted delivery, chemotherapy, and photothermal therapy into a single system. The physicochemical properties, biosafety, targeting capability, and therapeutic efficacy against PCa were systematically evaluated. Our results demonstrate a high degree of synergy between structural design and functional integration, highlighting the pronounced advantages of this nanoplatform for precision diagnosis and treatment of PCa.

Conventional small-molecule fluorescent probes, such as indocyanine green, predominantly operate in 700- to 900-nm NIR-I and suffer from aggregation-caused quenching, which severely limits tissue penetration depth and imaging signal-to-noise ratio [29,30]. In contrast, the PoMVT polymer synthesized in this study exhibits typical AIE characteristics, with an emission peak located in 1,080-nm NIR-II, which offers superior tissue penetration [31]. Notably, we introduced a unique “raisin-bun” structural design, which confers distinct optical advantages compared with conventional core–shell NPs [32]. Previous studies have shown that hydrophobic dyes directly embedded within lipid bilayers often exhibit unstable fluorescence or severe quenching due to spatial confinement and aggregation [33]. By encapsulating AIE clusters within the aqueous core of liposomes, our design not only exploits the AIE effect to achieve fluorescence enhancement at high local concentrations but also effectively suppresses tissue autofluorescence through an exceptionally large Stokes shift (>400 nm), thereby markedly improving imaging contrast and signal-to-noise ratio [34]. Collectively, this strategy overcomes the poor photostability of conventional organic dyes and enables superior deep-tumor imaging performance compared with traditional NIR-I probes [35].

Systemic administration of nanomaterials necessitates careful consideration of pharmacokinetics, biodistribution, and toxicity. Regarding quantitative biodistribution and toxicity, numerous vesicle- or liposome-based drug delivery systems have been reported to exhibit prolonged retention in the liver and spleen, while nucleic acid nanocarriers and small-molecule drug carriers may also pose potential hepatic and renal toxicities. In our study, the STEAP1 and PSMA dual-targeting strategy enabled AIE-MNPs/DOX@S/P-lip to begin enriching in tumor regions as early as 1 h postinjection, with peak accumulation observed at 6 to 12 h. Additionally, the NPs remained stable throughout a 6-d serum incubation period. Compared to nontargeted counterparts, the dual-targeted formulation significantly reduced liposomal uptake and clearance by the liver, spleen, and kidneys. Concerning long-term toxicity, since photothermal treatment was strictly confined to the local solid tumor, mice in the AIE-MNPs/DOX@S/P-lip treatment group showed no detectable damage to major organs, and hepatic and renal functions remained intact. Importantly, histopathological examination at 1 month postadministration confirmed the absence of pathological changes in major organs, indicating that the nanomaterial itself does not induce long-term toxicity. Furthermore, in the tumor rechallenge experiment, all mice in the AIE-MNPs/DOX@S/P-lip + 808-nm laser group survived with minimal tumor recurrence, supporting the favorable long-term safety profile of our therapeutic strategy.

With respect to targeting strategies, numerous studies have also constructed tumor-targeting nanodelivery systems through various approaches. For instance, Zhang et al. [36] developed a liposomal formulation modified with biotinylated GPC3 antibodies to target hepatocellular carcinoma. Masoudi et al. modified 2 distinct aptamers on the nanodelivery system to achieve breast cancer targeting [11,37]. In our study, we employed liposomes decorated with scFv selected through phage display screening for their targeting capability. Compared with antibodies, these scFvs possess a smaller molecular weight and lack nontargeting regions such as the Fc fragment; compared with other small-molecule ligands, they offer higher specificity. PSMA has been well established as one of the most representative molecular targets in PCa and has been widely employed in imaging probes and drug delivery systems. Meanwhile, STEAP1, another molecule highly expressed on the surface of PCa cells, has recently attracted increasing attention and is regarded as an important complementary target to PSMA [11,38]. However, most existing nanotherapeutic systems rely on single-target designs, which are insufficient to address the pronounced molecular heterogeneity of PCa [39]. In this study, both PSMA and STEAP1 antibodies were simultaneously conjugated onto the surface of the same nanocarrier. In vitro experiments demonstrated that in PSMA-low–expressing PC-3 cells, uptake mediated by PSMA-only targeting was significantly reduced compared with the dual-targeted formulation, whereas in 22Rv1 cells, the dual-targeted nanoplatform achieved the highest accumulation efficiency. These results strongly support the concept of “multi-target cooperative recognition”, indicating that simultaneous targeting of PSMA and STEAP1 enables broader coverage of heterogeneous tumor cell populations and effectively compensates for the limitations of single-target strategies. This approach opens a possible therapeutic pathway to the treatment of PSMA-negative or weakly PSMA-positive PCa patients [40,41].

Regarding the construction of multifunctional nanoplatforms, AIE luminogens and Fe₃O₄ MNPs are commonly incorporated to enable fluorescence imaging, photothermal therapy, magnetic hyperthermia, and MRI [12]. However, previous studies have largely emphasized the comprehensiveness of functional integration, while the relative contribution of each functional module to therapeutic outcomes has seldom been systematically evaluated [42]. Through a direct comparison of photothermal and magnetothermal effects, we found that under the material composition and external stimulation conditions employed in this study, photothermal therapy served as the dominant source of heat generation, whereas the contribution of magnetic hyperthermia was limited. Although some reports have suggested that iron oxide NPs can achieve efficient thermal ablation via magnetic hyperthermia, our results indicate that, within biologically safe dosage ranges, alternating magnetic fields alone did not induce cytotoxic temperatures [43]. This observation is consistent with recent thermodynamic studies showing that the specific absorption rate of small superparamagnetic NPs is inherently limited due to their reduced magnetic anisotropy and diminished hysteresis losses at the nanoscale, rather than achieving optimal heating efficiency [44]. Accordingly, rather than forcibly pursuing magnetothermal efficacy, we rationally utilized the magnetic component primarily for T_1_-weighted MRI enhancement and magnetic targeting. Experimental results confirmed that an external magnetic field markedly accelerated early NP accumulation at tumor sites (within 3 h). These findings do not diminish the value of the magnetic component but instead underscore its role as an auxiliary module for structural stabilization, imaging enhancement, and magnetically guided accumulation. Overall, this “magnetic navigation plus photothermal/chemotherapy” strategy appears more clinically translatable than approaches relying solely on magnetic hyperthermia.

At the mechanistic level, photothermal-chemotherapy synergism has been widely reported to enhance antitumor efficacy; however, most studies remain descriptive at the phenotypic level. In this work, transcriptomic sequencing was employed to further elucidate the underlying mechanisms of AIE-MNPs/DOX@S/P-lip. Differential gene expression analysis revealed significant alterations in genes associated with cell cycle regulation (e.g., PLK1 and SKA3), oxidative stress, and apoptosis. Notably, KEGG pathway enrichment and GO analyses highlighted changes in the intrinsic apoptotic signaling pathway and extracellular matrix organization. Combined with the experimentally observed elevation of intracellular ROS levels, we propose a “multimodal assault” mechanism: DOX disrupts DNA replication, leading to cell cycle arrest [45]. Simultaneously, Fe₃O₄ NPs may generate ROS, potentially inducing ferroptosis in PCa cells [46,47]. The photothermal effect further amplifies oxidative stress, ultimately resulting in irreversible cellular damage [48,49].

Despite these encouraging results, several limitations remain. First, the magnetothermal effect was not sufficiently pronounced under the current experimental conditions, and further optimization of magnetic core size, loading content, and alternating magnetic field parameters will be required to explore the feasibility of triple-modal photothermal-magnetothermal-chemotherapy synergy. In addition, the in vivo evaluation was limited to subcutaneous xenograft models, which do not fully reproduce the invasiveness and microenvironmental heterogeneity of clinical PCa. Future studies employing orthotopic PCa models are therefore warranted [50]. In addition, transcriptomic analysis provided only a preliminary insight into the molecular mechanisms, and further validation at the protein level and through functional assays is necessary. Detailed investigation of key differentially expressed genes, such as PLK1 and BTG2, and their downstream signaling pathways will be essential to elucidate the core molecular mechanisms underlying tumor cell eradication. Finally, the relatively high cost associated with large-scale production of dual-scFv antibodies discovered by phage display should also be considered [51].

In summary, the AIE-MNPs/DOX@S/P-lip nanoplatform developed in this study achieves integrated diagnosis and therapy with high efficacy and low toxicity in PCa models through the integration of multiple functionalities. Notably, this platform represents the first reported nanotheranostic system that integrates NIR-II/MRI dual-modal imaging and photothermal-chemo-immunotherapy specifically for PCa management, while simultaneously achieving STEAP1/PSMA dual-scFv targeting and synergistic photothermal-chemotherapy. This nanoplatform not only demonstrates potent tumor growth inhibition but also activates CD8^+^ T cells and macrophages to elicit potent antitumor immune responses. Consequently, this design provides new insights into the development of multifunctional theranostic nanodelivery systems and represents a promising strategy for precision immunotherapy of PCa.

## Methods

### Preparation of AIE-MNPs/DOX@S/P-lip

AIE-MNPs/DOX@S/P-lip were fabricated through a 4-step modular strategy. First, Fe₃O₄ MNPs were synthesized by coprecipitation: FeCl₂·4H₂O (0.017 mol, Klamar, China) and FeCl₃·6H₂O (0.029 mol, Klamar, China) were dissolved in 320 ml of purified water and sonicated until fully dispersed. Under N₂ protection, the mixture was magnetically stirred (600 rpm) at 80 °C for 60 min, followed by the rapid addition of 10 ml of NH₃·H₂O (25 wt %, Sinopharm Chemical Reagent Co., Ltd) and further reaction for 1 h. The final pH of the reaction system was maintained at approximately 10.5. Subsequent to cooling down to room temperature (RT), the solid was retrieved by centrifugation and exhaustively washed by deionized H_2_O and ethanol and lyophilized to obtain Fe₃O₄ MNPs. Next, 10 mg of the resulting Fe₃O₄ MNPs was redispersed in 10 ml of deionized water. An AIE-active photosensitizer, PoMVT, was successfully synthesized via Stille coupling polymerization of 4,7-dibromo-5,6-dioctyl-2,1,3-benzothiadiazole derivative (0.2 mmol) and 5,5′-bis(trimethylstannyl)-2,2′-bithiophene derivative (0.2 mmol), using tris(o-tolyl)phosphine (P(o-tol)₃) and tris(dibenzylideneacetone)dipalladium(0) (Pd₂(dba)₃) as catalysts in toluene at 100 °C for 24 h. The crude product was collected by filtration and further purified by Soxhlet extraction using methanol, n-hexane, and chloroform sequentially to eliminate catalyst residues and oligomers. The final polymer was recovered from the chloroform fraction and dried under vacuum, yielding PoMVT as a dark solid (Yield: 76%, M_n_: 25.0 kDa). PoMVT (2 mg, SuZhou Zhuoxinya Technology Co., Ltd, China) and Pluronic F-127 (20 mg, Aladdin) were dissolved in 1,000 μl of tetrahydrofuran (Aladdin) and introduced dropwise into the vigorously stirred Fe₃O₄ dispersion. The components were then stirred at RT for 120 min, magnetically collected and washed to yield AIE-decorated MNPs (AIE-MNPs). Liposomes were achieved via the thin-film hydration technique. Egg phosphatidylcholine (Aladdin, China), DSPE-PEG₂₀₀₀-COOH (Aladdin, China), cholesterol (Aladdin, China), and DSPE-PEG₂₀₀₀ (Aladdin, China) were dissolved in a 1:2 (v/v) methanol/chloroform solution system at a molar ratio of 3:2:0.2:0.3 and rotary evaporation to yield a thin lipid film. DOX (2 mg, Aladdin, China) and 2 ml of AIE-MNPs dispersion were added for hydration. The suspension underwent sequentially extrusion via 800- and 400-nm polycarbonate membranes, yielding homogeneous AIE-MNPs/DOX liposomes. Finally, dual-antibody targeting moieties were conjugated via EDC/NHS chemistry. Liposomes were activated in MES buffer (pH 5.5, MedChemExpress, China) containing 0.1 M EDC (Aladdin, China) and 0.7 M NHS (Aladdin, China) for 30 min. After concentration, 25 μg of anti-STEAP1 scFv and 25 μg of anti-PSMA scFv were incubated at 4 °C for 12 h. Unreacted sites were blocked by glycine (Aladdin, China), and the formulation was purified by dialysis (molecular weight cutoff: 100 kDa) to obtain AIE-MNPs/DOX@S/P liposomes. The final liposomes were resuspended in purified water (1,000 μg/ml) and saved at 4 °C.

### Characteristics of AIE-MNPs/DOX@S/P-lip

The structural identity of the AIE-active photosensitizer PoMVT was verified by ^1^H-nuclear magnetic resonance spectroscopy. The optical performance of PoMVT in the NIR-IIa window was assessed with UV–Vis spectroscopy and fluorescence spectroscopy, enabling optimization of excitation wavelength, emission filters, and exposure settings for mouse imaging. NIR-II fluorescence imaging was implemented using an NIR imaging system (Shanghai Artemis Intelligent Imaging Technology Co., Ltd, MARS-FAST) to evaluate the AIE characteristics. Morphology was observed by TEM, and elemental distribution was mapped by energy-dispersive x-ray spectroscopy. Hydrodynamic size and colloidal stability were quantified by nanoparticle tracking analysis (supported by Duolaimi Biotechnology with a ZetaView instrument), zeta-potential measurements (Malvern, Zetasizer Nano ZS), and dynamic light scattering. Powder x-ray diffraction was implemented to confirm the crystallinity of Fe₃O₄ magnetic cores (PANalytical, X’Pert PRO MPD). Molecular vibrational fingerprints were probed via Raman spectroscopy (Renishaw, inVia) and Fourier-transform infrared spectroscopy (Thermo Fisher Scientific, IS50), while surface chemistry and oxidation states were resolved by x-ray photoelectron spectroscopy. Direct visualization of dual-antibody spatial colocalization on the NP surface was achieved via direct stochastic optical reconstruction microscopy (Zeiss, Zeiss Elyra7). The samples underwent magnetic analysis via VSM using a Lake Shore 7404 instrument, and magnetothermal conversion efficiency was evaluated using a magnetic hyperthermia analyzer. Photothermal performance and stability were measured under 808-nm laser irradiation (1 W cm^−2^, spot size: 1.0 cm^2^, distance: 5 cm). Storage stability was monitored by tracking hydrodynamic diameter and PDI at pH 7.4 and 5.5, at 4 and −80 °C, over 168 h.

### Encapsulation rate, drug loading rate, and in vitro release assay

In vitro DOX-release profiles were acquired under distinct microenvironments. First, a calibration curve was established by testing the absorbance of serial DOX solutions at 480 nm in a quartz cuvette using a UV–Vis spectrophotometer. The encapsulation rate (EE %) and drug loading (DL %) were measured by standard curve: DL (%) = (total drug mass − unencapsulated drug mass)/total mass of drug-laden liposomes × 100 %. EE (%) = (total drug mass − unencapsulated drug mass)/total drug mass × 100%. Subsequently, 2 ml of AIE-MNPs/DOX@S/P-lip (2 mg/ml) was hermetically enclosed in a dialysis bag (molecular weight cutoff: 100 kDa) and immersed in 10 ml of PBS (pH 7.4 or 5.5) with or without NIR irradiation (808 nm, 1.0 W cm^−2^). The release experiments were conducted at 37 °C under gentle orbital shaking. At scheduled intervals, 2 ml of the external medium was removed and replaced with an equal volume of fresh PBS to sustain sink conditions. The amount of released DOX in aliquot was detected by UV–Vis spectrophotometry at 480 nm using the preestablished calibration equation. All data are represented as mean ± SD (*n* = 3).

### Photothermal performance and therapy of AIE-MNPs/DOX@S/P-lip

The photothermal conversion capability and therapeutic efficacy of AIE-MNPs/DOX@S/P-lip were evaluated in vitro and in vivo using an 808-nm NIR laser (fixed power density [*I*]: 1.0 W cm^−2^).

#### In vitro photothermal characterization

Aqueous dispersions of AIE-MNPs/DOX@S/P-lip at concentrations of 0, 0.05, 0.1, and 0.4 mg/ml were prepared in PBS (200 μl per sample) and placed in quartz cuvettes. Samples were irradiated for 600 s (10 min) with temperature recorded every 30 s using a calibrated thermocouple microprobe. Spatial temperature distribution was simultaneously monitored by infrared thermal imaging (A655sc). To determine photothermal conversion efficiency (η), the 400 μg/ml sample was irradiated to reach steady-state temperature (T_max), the laser was switched off, and the cooling curve was recorded every 30 s for 600 s. The η value was calculated using the energy balance method based on Newton’s law of cooling, as previously described (τ_s_ = 1.76 min, η = 67.4%). The η was calculated according to the equation: η = [*hS* (Tmax − Tsurr) − Qdis]/[*I* (1-10^-A808^)]. To determine the heat transfer coefficient (hS), the 400 μg/ml sample was heated to a steady-state temperature (Tmax = 72.1 °C) and then allowed to cool naturally. A dimensionless temperature parameter, θ = (T − Tsurr) / (Tmax − Tsurr), was introduced to linearize the cooling profile. The system time constant, τ_s_ = 1.76 min, was obtained by fitting the linear relationship between time and −lnθ. The hS value was subsequently derived from hS = mDCD / τ_s_, where mD and CD are the mass and heat capacity of the solvent, respectively. Using these parameters, the η was calculated to be 67.4%. Additionally, the composite maintained consistent peak temperatures over 4 ON/OFF irradiation cycles, demonstrating excellent photostability.

#### In vitro photothermal therapy

A total of 10^5^ PCa cells were seeded into each well of 6-well plates or glass-bottom dishes and incubated with AIE-MNPs/DOX@S/P-lip (10 μg/ml) for 6 h, and then cells were irradiated for 300 s while maintaining the culture temperature using an infrared temperature detector. Cell viability was detected by CCK-8 assay, and apoptosis was assessed by flow cytometry 24 h posttreatment.

#### In vivo photothermal therapy

Once subcutaneous xenografts attained approximately 150 mm^3^ in size, tumor-bearing mice received intravenous injection of AIE-MNPs/DOX@S/P-lip (3 mg/kg, 200 μl). Six hours after injection, the tumor area was exposed to irradiation with 808-nm laser for 600 s. Tumor surface temperature was monitored by infrared thermometry every 60 s. Tumor growth was monitored daily after photothermal therapy.

### Evaluation of the magnetothermal performance

#### Concentration-dependent magnetothermal response

To evaluate the magnetothermal conversion ability of AIE-MNPs/DOX@S/P-lip, we prepared suspensions at different dilutions (0, 50, 100, and 400 μg/ml) in deionized water, with each sample in a 1,500-μl centrifuge tube. These centrifuge tubes were positioned in the center of an alternating magnetic field generator (frequency: 450 kHz, field intensity: 25 mT, voltage: 120 to 160 V, current: 12 A). To capture real-time temperature data, a fiber optic temperature probe was introduced into the core of each sample, with readings taken every 30 s for 800 s. Deionized water served as a control to account for background heating. Temperature time data were used to plot temperature rise curves (ΔT-Time) and analyze the relationship between magnetothermal conversion efficiency and concentration.

#### Infrared thermal imaging analysis

Infrared thermography was employed to monitor temperature changes during the magnetothermal process. A suspension of AIE-MNPs/DOX@S/P-lip (400 μg/ml, 2 ml) was placed in a custom sample holder, with the thermal camera positioned 30 cm directly above. Under the same magnetic field conditions, thermograms were collected at predetermined temporal points (0, 300, 600, 1,200, 1,800, 2,400, 3,200, 4,000, and 4,800 s) to illustrate the uniformity and dynamic changes in temperature distribution.

#### Heating–cooling cycle stability test

To assess the thermal stability and reusability of the AIE-MNPs/DOX@S/P-lip, a heating–cooling cycle test was conducted. A suspension of AIE-MNPs/DOX@S/P-lip (400 μg/ml, 2 ml) was heated in the magnetic field for 5 min and subsequently cooled to RT without the field, completing 1 cycle. An infrared thermal camera monitored temperature changes throughout the process, recording the maximum temperature of each heating cycle and the cooling kinetics to evaluate the reproducibility of the magnetothermal effect.

### Cell culture

22Rv1 cells (CX0041) were purchased from Boster (Wuhan, China). LNCaP clone FGC (CL-0143) and PC-3 (CL-0185) were purchased from Procell (Wuhan). The mouse prostate carcinoma RM1 (SNL-122) and the C4-2 (SNL-160) cell lines were obtained from SUNNCELL (Wuhan). 22Rv1, LNCaP clone FGC, PC-3, and C4-2 cells were cultured in RPMI 1640 medium (KeyGEN, China) (containing 10% fetal bovine serum [FBS] [ABW, China, AB- FBS-1050], 100 U/ml penicillin, and 100 μg/ml streptomycin). RM1 cells were cultured in Dulbecco’s modified Eagle’s medium (KeyGEN, China) added with 10% FBS and 100 μg/ml streptomycin, and 100 U/ml penicillin. Cells were cultured at 37 °C and 5% CO_2_ in a saturated humidity incubator.

### Animal experiments

All mouse procedures were authorized by the Committee of Tongji Hospital (Approval No.: TJH-202312027). Subcutaneous neoplasms were induced in both C57BL/6 and BALB/c nude mouse strains. Mice aged 4 weeks were subjected to 7-d quarantine following arrival at the specific pathogen-free barrier facility for acclimatization. Mice (male, 4 to 6 weeks old) were supplied by Hunan SJA Laboratory Animal Co., Ltd. The living environment of the mice were kept at a temperature of ~25 °C with a 12-h light/dark cycle, with free access to standard food and water. The humane end points included tumor burden exceeding 1,500 mm^3^, animal weight loss exceeds 20% of normal animal weight, ulcer at tumor growth point, and sustained self-mutilation in animals.

#### Subcutaneous xenograft in BALB/c nude mice

22Rv1 cells were engineered to stably express luciferase (22Rv1-luc). Cells were cultured to 80% confluency, trypsinized, washed once with PBS, and resuspended in ice-cold PBS. Matrigel (ABW, Shanghai, China) was added at a 1:1 volume ratio to prepare a PBS–Matrigel cell suspension at a final concentration of 5 × 10^6^ cells/100 μl. For tumor inoculation, the flank of each BALB/c nude mouse was disinfected, and 100 μl of the cell suspension was injected subcutaneously using an insulin syringe.

#### Subcutaneous tumor in C57BL/6 mice

Murine PCa RM1 cells overexpressing human PSMA, STEAP1, and luciferase (RM1-STEAP1-PSMA-luc) were generated. Cells at 80% confluency were trypsinized, washed once with PBS, and resuspended in ice-cold PBS at a concentration of 1 × 10^6^ cells/100 μl. Tumors were established by subcutaneous injecting 100 μl of the cell suspension into the disinfected flank of C57BL/6 mice using an insulin syringe.

#### Tumor measurement

Mice were monitored daily for health status and tumor engraftment. Experiments were initiated when tumor volume reached approximately 100 mm^3^. Tumor length and width were measured using a caliper, and the tumor volume was calculated as (length × width^2^)/2. Additionally, tumor growth and size were monitored by detecting bioluminescence signal intensity. Specifically, mice bearing luciferase-expressing RM1 or 22RV1 tumors were intraperitoneally injected with potassium luciferin (100 μl per mouse, 14.3 mg/ml), and bioluminescence imaging was performed on an in vivo imaging system 10 min postinjection to monitor tumor size.

#### Direct cytotoxicity and photothermal therapy evaluation

Direct cytotoxicity and photothermal therapy were evaluated in tumor-bearing male BALB/c nude and C57BL/6 mice (*n* = 5 per group). Mice received tail vein injections of AIE-MNPs/DOX@S/P-lip or other control drugs (3 mg/kg, 200 μl) once every 3 d for 3 cycles. Immediately after intravenous drug administration, a 0.5-T neodymium disk was affixed to the tumor skin of each mouse for the first 10 min. For the photothermal therapy group, the tumor region was irradiated with an 808-nm laser (1.0 W cm^−2^, 10 min) 6 h postinjection, during which the surface temperature was maintained at 43 ± 0.5 °C. Body weight was monitored throughout the study.

### In vivo imaging

Tumor targeting and systemic distribution were evaluated by in vivo imaging. When tumors reached approximately 150 mm^3^, mice were randomized into treatment groups and injected intravenously via the tail vein with Cy5.5-labeled AIE-MNPs/DOX@S/P-lip and other regents (200 μl). For magnetic field enhancement, a neodymium magnet (field strength: 0.5 T) was positioned directly over the tumor site for 10 min postinjection. Whole-body fluorescence imaging was performed at predetermined time points (1, 3, 6, 12, 24, and 48 h) using an in vivo imaging system (AniView 100, Guangzhou BioLight Biotechnology, China). Mice were anesthetized with 2% isoflurane and placed supine on the heated platform. Fluorescence signals were acquired with excitation at 675 nm and emission at 740 nm. To delineate tumor boundaries, bioluminescence imaging of 22Rv1-luc tumors or RM1-STEAP1-PSMA-luc was conducted following intraperitoneal injection of D-luciferin (0.15 g/kg) (ShareBio, China). Image analysis and tumor-to-background ratios were calculated using Gv6000ProII software by region-of-interest quantification of fluorescence intensity in the tumor normalized to adjacent nontumor tissue.

At 48 h postinjection, mice were euthanized. Major organs and tumors were collected for ex vivo imaging using the same parameters. Fluorescence intensity for each organ was quantified and normalized.

### RNA sequencing and bioinformatics analysis

Total RNA was extracted from treated cells using TRIzol reagent (Invitrogen) according to the manufacturer’s protocol. RNA integrity was assessed using an Agilent Bioanalyzer 2100 system (RNA integrity number >7.0 required for downstream analysis). Library preparation and transcriptome sequencing were performed by OE Biotech (Shanghai, China) using the NEBNext Ultra RNA Library Prep Kit for Illumina and sequenced on a NovaSeq 6000 platform (150-bp paired-end reads). The raw sequencing datasets generated in this study have been deposited in Figshare and are accessible at https://doi.org/10.6084/m9.figshare.31156933.

Raw sequencing data were processed and analyzed in R (v4.3.0). Adapter trimming and quality control were performed using TrimGalore. Clean reads were aligned to the reference genome (hg38 for human samples) using HISAT2. Gene-level quantification was conducted with featureCounts, and differential expression analysis was performed using DESeq2. Genes with |log₂ fold change| > 0.58 (corresponding to fold change >1.5) and adjusted *P* value <0.05 were considered statistically significant. GO and KEGG pathway enrichment analyses were performed using clusterProfiler. For GSEA, all genes were ranked by the log₂ fold change values derived from DESeq2. Preranked GSEA was conducted using the clusterProfiler::GSEA function, with the KEGG pathway gene sets (from the KEGG database) and the Reactome pathway gene sets (from the Reactome database). Enriched gene sets were identified using 1,000 permutations, and adjusted *P* values (false discovery rate) < 0.25 were considered statistically significant, in line with standard GSEA recommendations. Volcano plots, heatmaps, and GSEA enrichment plots were generated using ggplot2 and pheatmap packages.

### Western blot

Total protein from cells or tumor tissues was extracted with radioimmunoprecipitation assay buffer containing protease inhibitors (Epizyme, China). Equal protein samples were resolved by sodium dodecyl sulfate-polyacrylamide gel electrophoresis and blotted onto polyvinylidene difluoride membranes (Vazyme, China) using electrophoresis and transfer buffers (Servicebio, China). After blocking with 5% skimmed milk, membranes were incubated overnight with primary antibodies (detailed information listed in Table S2) at 4 °C and corresponding secondary antibodies at RT. Detected proteins included p53, p21, LC3B-I, LC3B-II, P62, RIP1, p-RIP1, MLKL, p-MLKL, BAX, BCL2, cleaved caspase-3, HMGB1, GPX4, ACSL4, and SLC7A11; glyceraldehyde phosphate dehydrogenase was used as the internal loading control. Target bands were visualized via chemiluminescence (Asure Biosystems 600).

### Lentiviral infection

The CMV-luc-Neo lentivirus was designed and synthesized by Genomeditech (Shanghai, China), and overexpression of STEAP1 and PSMA lentivirus was constructed by WZ Biosciences Inc (Shandong, China). Cells were transduced with lentiviral vectors or empty vector controls, selected using antibiotics, and analyzed for mRNA and protein expression by reverse transcription quantitative polymerase chain reaction and Western blotting, respectively.

### Immunofluorescence staining and confocal microscopy

For cellular F-actin staining, 22Rv1 cells were fixed with 4% paraformaldehyde for 15 min. F-actin was labeled using phalloidin (1:500, 1 h, protected from light; Beyotime Biotechnology), and cell nuclei were counterstained with 4′,6-diamidino-2-phenylindole (DAPI) (1:1,000, 10 min; Servicebio). After antifade mounting, fluorescence images were collected via confocal microscopy (LSM900 Airyscan).

For tissue immunofluorescence detection, 5-μm paraffin sections were dewaxed in xylene, rehydrated with graded ethanol, and rinsed with PBS. Antigen retrieval was performed in EDTA buffer (RC03, Shanghai Ruchuang Bio) via microwave treatment. Sections were permeabilized with 0.1% Triton X-100 for 15 min and blocked with 1.5% bovine serum albumin (BSA) for 60 min at RT. After overnight incubation with primary antibodies (Table S2) at 4 °C, sections were incubated with fluorophore-conjugated secondary antibodies (1:500, RC0086-23/34, Shanghai Ruchuang Bio) at 37 °C for 60 min. Following DAPI nuclear staining and neutral balsam mounting, whole-slide multichannel images were acquired with a Pannoramic MIDI scanner, and quantitative analysis was performed using CaseViewer software.

Multiplex immunofluorescence staining of ICD biomarkers (HMGB1 and CRT) was performed on drug-treated 22Rv1 cells with or without photothermal therapy using a commercial tyramide signal amplification fluorescence staining kit (RK50026P, ABClonal). After 6 h of photothermal treatment, cells were fixed with 4% paraformaldehyde. The samples were sequentially subjected to peroxidase and BSA blocking, overnight primary antibody incubation (Table S2), and horseradish peroxidase-conjugated secondary antibody incubation. Tyramide signal amplification was conducted with TYR-690 for HMGB1 and TYR-520 for CRT, and antibody stripping buffer was used for elution between staining cycles. Finally, nuclei were counterstained with DAPI, and samples were mounted with antifade medium for confocal imaging.

### Immunohistochemistry

Consecutive 5-μm sections were dewaxed, rehydrated, and subjected to antigen retrieval as above. Endogenous peroxidase was blocked with 3% H₂O₂ (Sinopharm) for 25 min at RT. After 3% BSA blocking, sections were incubated for 12 h at 4 °C with Ki-67 antibody (1:8,000, 27309-1-AP, Proteintech, China). Horseradish peroxidase-conjugated secondary antibody (RCA054, Shanghai Ruchuang Bio) was applied for 1 h at RT, followed by 3,3'-diaminobenzidine chromogen (Shanghai Ruchuang Bio, RC062) until brown precipitate appeared. Nuclei were counterstained with hematoxylin (RC0060, Shanghai Ruchuang Bio). Staining was evaluated under a BX53 microscope (Olympus, Japan) by 2 blinded pathologists.

### Transmission electron microscopy

For high-resolution uptake imaging, 22Rv1 cells were seeded in 10-cm dishes, treated with indicated formulations for 6 h, and immediately fixed with 2.5% glutaraldehyde in 100 mM phosphate buffer for 120 min at RT. After 3 rinses with 100 nM phosphate buffer, cells were scraped off with a cell scraper (45° angle, unidirectional), pelleted at 2,000 rpm for 5 min, and fixed in 1% osmium tetroxide at 4 °C for 120 min. The pellet was then dehydrated through a graded ethanol series (30%, 50%, 70%, 90%, and 100%, 10 min each), impregnated with Epon-Araldite resin and cured at 60 °C for 2 d. Semithin sections (1 μm) were cut. Ultrathin sections (70 nm) were placed on grids, stained with uranyl acetate (3%) and lead citrate (2.7%), and examined by TEM.

### In vivo NIR-II imaging and MRI

#### In vivo NIR-II imaging

The tumor NIR-II imaging capability of AIE-MNPs/DOX@S/P-lip was evaluated in nude mice bearing 22Rv1-luc xenografts using an IVIS Spectrum system equipped with a NIR-II channel (1,000 to 1,700 nm, PerkinElmer, USA). Following intravenous injection of the respective probes via the tail vein, mice in the AIE-MNPs/DOX@S/P-lip + magnetic field group had a 0.5-T neodymium disk fixed on the tumor skin for the first 10 min. Tumor location was first confirmed by bioluminescence imaging. NIR-II imaging was then performed at 1, 3, 6, 12, 24, 48, and 72 h postinjection. Major organs and tumors were harvested immediately for ex vivo NIR-II imaging.

#### Magnetic resonance imaging

MRI was performed concurrently with the NIR-II imaging study. Briefly, 24 h after drug administration and following the in vivo NIR-II scan, mice were transferred to a dedicated small-animal MRI system (Bruker, BioSpec7.0T) for T_1_-weighted imaging to evaluate the contrast enhancement effects of the probes within the tumor region.

### Flow cytometry of tumor tissues

Tumor tissue samples were excised from mice and placed in ice-cold serum-free basal medium enzymatically supplemented with hyaluronidase, deoxyribonuclease I, and collagenase (1 mg/ml). Tissues were minced with surgical scissors and incubated at RT for 120 min to ensure digestion. Single cells were subjected to gradient centrifugation to separate tumor and immune cells. Immune cells were collected, subjected to red blood cell lysis, centrifuged, and stained with Zombie viability dye for 25 min at RT. After washing, cells were labeled with surface markers (antibody details are provided in Table S2), then fixed, permeabilized, and stained intracellularly. Finally, stained cells were resuspended in PBS, filtered again through a 200-μm mesh, and evaluated by means of flow cytometry (Beckman, USA). The gating strategy details are provided in Fig. S16A to C.

### In vivo tumor rechallenge and memory T cell analysis

Mice were inoculated subcutaneously in the right flank with 1 × 10^6^ RM1-STEAP1-PSMA-luc cells. When tumors reached 50 to 100 mm^3^, animals were randomized into 4 groups (*n* = 10): Naive control (G1); PBS (G2); AIE-MNPs/DOX@S/P-lip (G3); and AIE-MNPs/DOX@S/P-lip plus 808-nm laser irradiation (G4). All mice were divided into 2 cohorts (*n* = 5) after primary treatment. Cohort 1 (tumor rechallenge): At day 22, mice were rechallenged by subcutaneous injection of 1 × 10^6^ RM1-STEAP1-PSMA-luc cells into the contralateral flank. Tumor growth and survival were monitored. Cohort 2 (immunological memory assessment): At 2 weeks posttreatment (day 14), mice were euthanized and lymph nodes, spleens, and peripheral blood were harvested for flow cytometry analysis of memory T cell populations. Single-cell suspensions were generated by gentle mechanical dissociation through a 70-μm cell strainer, and erythrocytes were removed using red blood cell lysis buffer. Spleens were photographed and weighed before processing, and the splenic index was recorded. Cells were incubated with a fixable viability dye, followed by surface staining with anti-CD3, anti-CD8a, anti-CD44, and anti-CD62L antibodies. Within the live CD3^+^CD8^+^ gate, T_naive_, T_CM_, and T_EM_ cells were defined as CD44^−^CD62L^+^, CD44^+^CD62L^+^, and CD44^+^CD62L^−^, respectively.

### Ex vivo antigen restimulation and intracellular cytokine staining

Splenocytes (2 × 10^6^ cells per well) were restimulated with tumor antigen (RM1-STEAP1-PSMA-luc cell lysate) for 6 h, with brefeldin A added at the start of culture to inhibit cytokine secretion. Cells were surface-stained for CD3 and CD8a, fixed and permeabilized, and stained intracellularly for IFN-γ and perforin. The frequencies of IFN-γ^+^ and perforin^+^ cells were determined within the CD8^+^ gate.

### Statistical analysis

Quantitative data are shown as means ± SD. Normality was tested with Shapiro–Wilk. Two-group comparisons used 2-tailed Student *t* test (normal distribution) or Mann–Whitney U test (non-normal). Multigroup comparisons used 1-way analysis of variance (ANOVA) with Tukey’s post hoc test. Survival was analyzed by log-rank (Mantel–Cox) test. Statistics were done in GraphPad Prism v10.0 (GraphPad Software, USA). *P* < 0.05 was considered significant.

## Data Availability

The authors declare that all data supporting the findings of this study are available within the article and its Supplementary Materials.

## References

[1] Bergengren O, Pekala KR, Matsoukas K, Fainberg J, Mungovan SF, Bratt O, Bray F, Brawley O, Luckenbaugh AN, Mucci L, et al. 2022 Update on prostate cancer epidemiology and risk factors—A systematic review. Eur Urol. 2023;84(2):191–206. 37202314 10.1016/j.eururo.2023.04.021 PMC10851915

[2] Chakrabarti D, Albertsen P, Adkins A, Kishan A, Murthy V, Parker C, Pathmanathan A, Reid A, Sartor O, Van As N, et al. The contemporary management of prostate cancer. CA Cancer J Clin. 2025;75(6):552–586. 40572035 10.3322/caac.70020 PMC12593286

[3] Kirby M, Hirst C, Crawford ED. Characterising the castration-resistant prostate cancer population: A systematic review. Int J Clin Pract. 2011;65(11):1180–1192. 21995694 10.1111/j.1742-1241.2011.02799.x

[4] Candelieri-Surette D, Lee J, Lynch JA, Chang N-CN, Nelson TJ, Teerlink CC, Pimentel C, Schoen MW, Berlowitz D. Epidemiology of metastatic castration-resistant prostate cancer in veterans nationwide. J Natl Compr Cancer Netw. 2025;23(8):307–313. 10.6004/jnccn.2025.7032 40659047

[5] Soon YY, Marschner IC, Schou M, Hofman MS, Emmett L, Davis ID, Stockler MR, Martin AJ. Lu-177 PSMA vs comparator treatments and survival in metastatic castration-resistant prostate cancer. JAMA Netw Open. 2024;7(9): Article e2433863. 39287944 10.1001/jamanetworkopen.2024.33863 PMC11409154

[6] de Bono J, Mateo J, Fizazi K, Saad F, Shore N, Sandhu S, Chi KN, Sartor O, Agarwal N, Olmos D, et al. Olaparib for metastatic castration-resistant prostate cancer. N Engl J Med. 2020;382(22):2091–2102. 32343890 10.1056/NEJMoa1911440

[7] Cheng Z, Li M, Dey R, Chen Y. Nanomaterials for cancer therapy: Current progress and perspectives. J Hematol Oncol. 2021;14(1):85. 34059100 10.1186/s13045-021-01096-0 PMC8165984

[8] Zhu J, Peng L, Jehan S, Wang H, Chen X, Zhao S, Zhou W. Activable photodynamic DNA probe with an “AND” logic gate for precision skin cancer therapy. Research. 2024;7:0295. 38269029 10.34133/research.0295 PMC10807844

[9] Wang H-P, Chen X, Qi Y-L, Huang L-W, Wang C-X, Ding D, Xue X. Aggregation-induced emission (AIE)-guided dynamic assembly for disease imaging and therapy. Adv Drug Deliv Rev. 2021;179: Article 114028. 34736987 10.1016/j.addr.2021.114028

[10] Xu W, Jian D, Yang H, Wang W, Ding Y. Aggregation-induced emission: Application in diagnosis and therapy of hepatocellular carcinoma. Biosens Bioelectron. 2024;266: Article 116722. 39232431 10.1016/j.bios.2024.116722

[11] Sun J-X, Xia Q-D, Xu J-Z, An Y, Ma S-Y, Xu J-Y, Xiang J-C, Liu C-Q, Xu M-Y, Zhang S-H, et al. A novel prostate cancer-specific fluorescent probe based on extracellular vesicles targeting STEAP1 applied in fluorescence guided surgery. J Control Release. 2025;380:199–218. 39894263 10.1016/j.jconrel.2025.01.079

[12] Chua MH, Chin KLO, Loh XJ, Zhu Q, Xu J. Aggregation-induced emission-active nanostructures: Beyond biomedical applications. ACS Nano. 2023;17(3):1845–1878. 36655929 10.1021/acsnano.2c10826

[13] Dadfar SM, Roemhild K, Drude NI, von Stillfried S, Knüchel R, Kiessling F, Lammers T. Iron oxide nanoparticles: Diagnostic, therapeutic and theranostic applications. Adv Drug Deliv Rev. 2019;138:302–325. 30639256 10.1016/j.addr.2019.01.005 PMC7115878

[14] Sun J-X, Xu J-Z, An Y, Ma S-Y, Liu C-Q, Zhang S-H, Luan Y, Wang S-G, Xia Q-D. Future in precise surgery: Fluorescence-guided surgery using EVs derived fluorescence contrast agent. J Control Release. 2023;353:832–841. 36496053 10.1016/j.jconrel.2022.12.013

[15] Roberts MJ, Maurer T, Perera M, Eiber M, Hope TA, Ost P, Siva S, Hofman MS, Murphy DG, Emmett L, et al. Using PSMA imaging for prognostication in localized and advanced prostate cancer. Nat Rev Urol. 2023;20(1):23–47. 36473945 10.1038/s41585-022-00670-6

[16] Wang L, Lu C, Wang X, Wu D. PSMA targeted therapy: From molecular mechanisms to clinical breakthroughs in castration-resistant prostate cancer. Eur J Med Chem. 2025;296: Article 117829. 40466338 10.1016/j.ejmech.2025.117829

[17] Boixareu C, Taha T, Venkadakrishnan VB, de Bono J, Beltran H. Targeting the tumour cell surface in advanced prostate cancer. Nat Rev Urol. 2025;22(9):569–589. 40169837 10.1038/s41585-025-01014-w PMC12954637

[18] Bakht MK, Beltran H. Biological determinants of PSMA expression, regulation and heterogeneity in prostate cancer. Nat Rev Urol. 2025;22(1):26–45. 38977769 10.1038/s41585-024-00900-z PMC11841200

[19] Wang Y, Wang Y, Ci X, Choi SYC, Crea F, Lin D, Wang Y. Molecular events in neuroendocrine prostate cancer development. Nat Rev Urol. 2021;18(10):581–596. 34290447 10.1038/s41585-021-00490-0 PMC10802813

[20] Gan Q, Cui K, Cao Q, Zhang N, Yang M-F, Yang X. Development of a ^18^F-labeled bicyclic peptide targeting EphA2 for molecular imaging of PSMA-negative prostate cancer. J Med Chem. 2023;66(21):14623–14632. 37908059 10.1021/acs.jmedchem.3c01135

[21] Bhatia V, Kamat NV, Pariva TE, Wu L-T, Tsao A, Sasaki K, Sun H, Javier G, Nutt S, Coleman I, et al. Targeting advanced prostate cancer with STEAP1 chimeric antigen receptor T cell and tumor-localized IL-12 immunotherapy. Nat Commun. 2023;14(1):2041. 37041154 10.1038/s41467-023-37874-2 PMC10090190

[22] Rezaei B, Yari P, Sanders SM, Wang H, Chugh VK, Liang S, Mostufa S, Xu K, Wang J-P, Gómez-Pastora J, et al. Magnetic nanoparticles: A review on synthesis, characterization, functionalization, and biomedical applications. Small. 2024;20(5): Article e2304848. 37732364 10.1002/smll.202304848

[23] Ko MJ, Min S, Hong H, Yoo W, Joo J, Zhang YS, Kang H, Kim D-H. Magnetic nanoparticles for ferroptosis cancer therapy with diagnostic imaging. Bioact Mater. 2024;32:66–97. 37822917 10.1016/j.bioactmat.2023.09.015 PMC10562133

[24] Saadat M, Manshadi MKD, Mohammadi M, Zare MJ, Zarei M, Kamali R, Sanati-Nezhad A. Magnetic particle targeting for diagnosis and therapy of lung cancers. J Control Release. 2020;328:776–791. 32920079 10.1016/j.jconrel.2020.09.017 PMC7484624

[25] Lin Y, Qiao J, Sun Y, Dong H. The profound review of Fenton process: What’s the next step? J Environ Sci. 2025;147:114–130. 10.1016/j.jes.2023.10.005 39003034

[26] Zhao H, Yu J, Zhang R, Chen P, Jiang H, Yu W. Doxorubicin prodrug-based nanomedicines for the treatment of cancer. Eur J Med Chem. 2023;258: Article 115612. 37441851 10.1016/j.ejmech.2023.115612

[27] Li N, Wang Y, Li Y, Zhang C, Fang G. Recent advances in photothermal therapy at near-infrared-II based on 2D MXenes. Small. 2024;20(6): Article e2305645. 37775938 10.1002/smll.202305645

[28] Croft M, Salek-Ardakani S, Ware CF. Targeting the TNF and TNFR superfamilies in autoimmune disease and cancer. Nat Rev Drug Discov. 2024;23(12):939–961. 39448880 10.1038/s41573-024-01053-9

[29] Cassinotti E, Al-Taher M, Antoniou SA, Arezzo A, Baldari L, Boni L, Bonino MA, Bouvy ND, Brodie R, Carus T, et al. European Association for Endoscopic Surgery (EAES) consensus on Indocyanine Green (ICG) fluorescence-guided surgery. Surg Endosc. 2023;37(3):1629–1648. 36781468 10.1007/s00464-023-09928-5 PMC10017637

[30] Ma S, Sun J, Xu J, An Y, Xu M, Liu C, Zhang S, Miao L, Zhong X, Zeng N, et al. The diagnostic performance of indocyanine green for the sentinel node biopsy in prostate cancer: A systematic review and meta-analysis. Asian J Urol. 2025;12(1):1–14. 39990074 10.1016/j.ajur.2024.07.001 PMC11840311

[31] Zhang Z, Du Y, Shi X, Wang K, Qu Q, Liang Q, Ma X, He K, Chi C, Tang J, et al. NIR-II light in clinical oncology: Opportunities and challenges. Nat Rev Clin Oncol. 2024;21(6):449–467. 38693335 10.1038/s41571-024-00892-0

[32] Wang J, Zhang Y, Xu X, Bao M. Oxygen vacancy-rich Ni-CeO_2_ heterojunction catalyst for hydrogenating halogenated nitroarenes with high activity and selectivity. ACS Appl Mater Interfaces. 2023;15(6):8149–8156. 36637974 10.1021/acsami.2c21272

[33] Dai J, Dong X, Wang Q, Lou X, Xia F, Wang S. PEG-polymer encapsulated aggregation-induced emission nanoparticles for tumor theranostics. Adv Healthc Mater. 2021;10(24): Article e2101036. 34414687 10.1002/adhm.202101036

[34] Likhotkin I, Lincoln R, Bossi ML, Butkevich AN, Hell SW. Photoactivatable large stokes shift fluorophores for multicolor nanoscopy. J Am Chem Soc. 2023;145(3):1530–1534. 36626161 10.1021/jacs.2c12567 PMC9880998

[35] Zhu S, Yung BC, Chandra S, Niu G, Antaris AL, Chen X. Near-infrared-II (NIR-II) bioimaging via off-peak NIR-I fluorescence emission. Theranostics. 2018;8(15):4141–4151. 30128042 10.7150/thno.27995 PMC6096392

[36] Zhang J, Luo X, Yang X, Li H, Jiang Q, Yang Y, Luo M, Ma Z, He P, Feng L, et al. Ultrasound-responsive nanodelivery system of GPC3-targeting and sonosensitizer for visualized hepatocellular carcinoma therapy. Int J Nanomedicine. 2024;19:7015–7031. 39011387 10.2147/IJN.S470847 PMC11249105

[37] Masoudi M, Taghdisi SM, Hashemitabar G, Abnous K. Targeted co-delivery of FOXM1 aptamer and DOX by nucleolin aptamer-functionalized pH-responsive biocompatible nanodelivery system to enhance therapeutic efficacy against breast cancer: In vitro and in vivo. Drug Deliv Transl Res. 2024;14(6):1535–1550. 38161196 10.1007/s13346-023-01495-5

[38] Nolan-Stevaux O, Li C, Liang L, Zhan J, Estrada J, Osgood T, Li F, Zhang H, Case R, Murawsky CM, et al. AMG 509 (Xaluritamig), an anti-STEAP1 XmAb 2+1 T-cell redirecting immune therapy with avidity-dependent activity against prostate cancer. Cancer Discov. 2024;14(1):90–103. 37861452 10.1158/2159-8290.CD-23-0984

[39] Sidorenko V, Tobi A, Sugahara KN, Teesalu T. Peptide-targeted nanoparticles for tumor therapy. J Control Release. 2025;387: Article 114195.40925419 10.1016/j.jconrel.2025.114195

[40] Sayar E, Patel RA, Coleman IM, Roudier MP, Zhang A, Mustafi P, Low J-Y, Hanratty B, Ang LS, Bhatia V, et al. Reversible epigenetic alterations mediate PSMA expression heterogeneity in advanced metastatic prostate cancer. JCI Insight. 2023;8(7): Article e162907. 36821396 10.1172/jci.insight.162907 PMC10132157

[41] Luo Y, Yang H, Zhou Y-F, Hu B. Dual and multi-targeted nanoparticles for site-specific brain drug delivery. J Control Release. 2020;317:195–215. 31794799 10.1016/j.jconrel.2019.11.037

[42] Chen Y, Sun H, Li Y, Han X, Yang Y, Chen Z, Zhao X, Qian Y, Liu X, Zhou F, et al. Magnetic nanomaterials for hyperthermia-based therapy and controlled drug delivery. Bioact Mater. 2025;53:591–629. 40761549 10.1016/j.bioactmat.2025.07.033 PMC12320187

[43] Chu M, Shao Y, Peng J, Dai X, Li H, Wu Q, Shi D. Near-infrared laser light mediated cancer therapy by photothermal effect of Fe_3_O_4_ magnetic nanoparticles. Biomaterials. 2013;34(16):4078–4088. 23465836 10.1016/j.biomaterials.2013.01.086

[44] Gavilán H, Avugadda SK, Fernández-Cabada T, Soni N, Cassani M, Mai BT, Chantrell R, Pellegrino T. Magnetic nanoparticles and clusters for magnetic hyperthermia: Optimizing their heat performance and developing combinatorial therapies to tackle cancer. Chem Soc Rev. 2021;50(20):11614–11667. 34661212 10.1039/d1cs00427a

[45] Mattioli R, Ilari A, Colotti B, Mosca L, Fazi F, Colotti G. Doxorubicin and other anthracyclines in cancers: Activity, chemoresistance and its overcoming. Mol Aspects Med. 2023;93: Article 101205. 37515939 10.1016/j.mam.2023.101205

[46] Neyens E, Baeyens J. A review of classic Fenton’s peroxidation as an advanced oxidation technique. J Hazard Mater. 2003;98(1–3):33–50. 12628776 10.1016/s0304-3894(02)00282-0

[47] Ye Z, Xie B, Tao Y, Xiao D. Mechanism of ferroptosis and its role in disease development. Int J Biol Sci. 2025;21(12):5328–5360. 40959280 10.7150/ijbs.102859 PMC12435488

[48] Hong E, Hyun H, Lee H, Jung E, Lee D. Acid-sensitive oxidative stress inducing and photoabsorbing polysaccharide nanoparticles for combinational anticancer therapy. Int J Pharm. 2020;574: Article 118893. 31765773 10.1016/j.ijpharm.2019.118893

[49] Overchuk M, Weersink RA, Wilson BC, Zheng G. Photodynamic and photothermal therapies: Synergy opportunities for nanomedicine. ACS Nano. 2023;17(9):7979–8003. 37129253 10.1021/acsnano.3c00891 PMC10173698

[50] Sailer V, von Amsberg G, Duensing S, Kirfel J, Lieb V, Metzger E, Offermann A, Pantel K, Schuele R, Taubert H, et al. Experimental in vitro, ex vivo and in vivo models in prostate cancer research. Nat Rev Urol. 2023;20(3):158–178. 36451039 10.1038/s41585-022-00677-z

[51] Guliy OI, Evstigneeva SS, Dykman LA. Recombinant antibodies by phage display for bioanalytical applications. Biosens Bioelectron. 2023;222: Article 114909. 36462427 10.1016/j.bios.2022.114909

