# Supplementary material for: Magnetically Navigated and PSMA/STEAP1 Dual-Targeted Nanoliposomes for NIR-II/MRI Imaging with Photothermal-Chemo-Immunotherapy for Prostate Cancer
Source: Research (Wash D C). 2026 Aug 14;9:1375. doi: 10.34133/research.1375 (PMC13473716; doi:10.34133/research.1375)
Supplement: Supplementary file 1 [file research.1375.f1.docx]

**Supplementary Materials**

**Title: Magnetically navigated and PSMA/STEAP1 dual-targeted nanoliposomes for NIR-II/MRI imaging with photothermal-chemo-immunotherapy for prostate cancer**

**Author list:** Jian-Xuan Sun^1^* , Ye-An^2^*, Si-Han Zhang^1^*, Jin-Zhou Xu^1^*, Maimaiti Abudureyimu^1^, Jing-Yu Xu^1^, Yi-Fan Huang^1^, Yu-Xuan Yang^1^, Mei-Cheng Liu^1^, Shao-Gang Wang^1#^, Qi-Dong Xia^1#^.

**Author affiliations and footnotes:**

***Co-first authorship:** These authors contributed equally to this work.

**#Co-correspondence authorship:** These authors contributed equally to this work.

1. Department and Institute of Urology, Tongji Hospital, Tongji Medical College, Huazhong University of Science and Technology, Wuhan, China.
2. Department of Urology, The First People's Hospital of Yunnan Province, Kunming University of Science and Technology Affiliated Hospital, Kunming, China.

**Contact information:**

Qi-Dong Xia, Department and Institute of Urology, Tongji Hospital, Tongji Medical College, Huazhong University of Science and Technology, Wuhan, P.R. China.

E-mail: qidongxia_md@163.com Telephone numbers: +86-027-83663460.

Shao-Gang Wang, Department and Institute of Urology, Tongji Hospital, Tongji Medical College, Huazhong University of Science and Technology, Wuhan, P.R. China.

E-mail: sgwangtjm@163.com Telephone numbers: +86-027-83663460.

**Figure S1. EDS-Mapping reveals the spatial distribution of each component in the nanocomposite.** Carbon is broadly distributed, confirming the presence of organic moieties (lipids, PEG, AIE photosensitizer, DOX). Oxygen originates mainly from Fe₃O₄, PEG ether linkages, and phospholipid ester groups. Iron appears as highly localized dots that perfectly overlap with the magnetic cores seen in TEM, verifying successful encapsulation. Nitrogen signal arises from phosphatidylcholine quaternary ammonium, DOX amino groups, and surface-conjugated antibodies. Phosphorus, characteristic of phospholipids, is evenly distributed, attesting to an intact bilayer. Weak but detectable sulfur implies successful antibody coupling via disulfide bonds. (Scale bars, 50 nm).

**Figure S2. Fine analysis of chemical valence states for core elements.**

**Figure S3. The Photothermal and Magnetothermal Effects of AIE-MNPs/DOX@S/P-lip. (A)** Cooling curves post heating. **(B)** Linear fitting of the sample cooling curve. **(C)** Cycling stability experiment confirming the photothermal stability and reusability of the material. **(D)** Effects of different lipid/drug (L/D) mass ratios on the encapsulation efficiency (EE) and drug loading content (DL) of AIE-MNPs/DOX@S/P-lip.

**Figure S4. *In Vitro* Cellular uptake of AIE-MNPs/DOX@S/P-lip. (A–C)** Time-dependent cellular uptake of AIE-MNPs/DOX@S/P-lip by 22Rv1 **(A)**, PC-3 **(B)**, and RM1 **(C)** cells.

**Figure S5. *In Vitro* Cytotoxicity of AIE-MNPs/DOX@S/P-lip. (A–G)** Cytotoxicity evaluated by CCK-8 assay in 22Rv1 **(A)**, RM1 **(B)**, PC-3 **(C)**,	C4-2 **(D, E)**, and LNCaP **(F, G)** cells after treatment 24h or 48h with AIE-MNPs/DOX@S/P-lip (0–40 μg/mL). **(H–L)** Cytotoxicity evaluated by CCK-8 assay in 22Rv1 **(H)**, RM1 **(I)**, PC-3 **(J)**, PC-3-STEAP1 **(K)**, and PC-3-PSMA **(L)** cells after 48-hour treatment with AIE-MNPs/DOX@S/P-lip (0–40 μg/mL). Data are presented as mean ± SD from n = 6 independent experiments (ns: no significance, **P < 0.01, ***P < 0.001, ****P < 0.0001).

**Figure S6. AIE-MNPs/DOX@S/P-lip Induces Apoptosis in Prostate Cancer Cells. (A–C)** Apoptosis assessed by flow cytometry in 22Rv1 **(A)**, PC-3 **(B)**, and RM1 **(C)** cells after 48-hour treatment with AIE-MNPs/DOX@S/P-lip (0–20 μg/mL).Data are presented as mean ± SD from n = 3 independent experiments (ns: no significance, **P < 0.01, ***P < 0.001, ****P < 0.0001).

**Figure S7. Transwell Migration and Colony Formation Inhibition by AIE-MNPs/DOX@S/P-lip in Prostate Cancer Cells. (A)** Inhibitory effect on cell migration measured by transwell assay in 22Rv1, PC-3, and RM1 cells after 48-hour treatment with AIE-MNPs/DOX@S/P-lip (0–20 μg/mL). **(B)** Inhibitory effect on colony formation in 22Rv1, PC-3, and RM1 cells treated with AIE-MNPs/DOX@S/P-lip (0–20 μg/mL). Each experiment was performed with three independent biological replicates for each group. Data are presented as mean±standard deviation. ns: no significance, *P < 0.05, **P < 0.01, ***P < 0.001, ****P < 0.0001.

**Figure S8. In Vivo Antitumor Efficacy and Safety of AIE-MNPs/DOX@S/P-lip.** (A) The animal experimental procedure. (B) The tumor growth curves (n = 5). (C) Ex vivo images of tumors after mouse sacrifice (n = 5). (D-E) The comparison of tumor volume (D) and weight (E) after mouse sacrifice (n = 5). (F) The body weight of mouse after drug administration (n = 5). (G-J) The liver and kidney function analysis of mice after drug administration. (K) The H&E staining of major organs (heart, liver, spleen, lung, and kidney) from BALB/c nude mice. Data are presented as mean±standard deviation. ns: no significance, *P < 0.05, **P < 0.01, ***P < 0.001, ****P < 0.0001. (Scale bars, 50 μm)

**Figure S9. *In Vivo* and *in V*itro Safety and Pharmacokinetics of AIE-MNPs/DOX@S/P-lip. (A-B)** Hemolysis experiment after AIE-MNPs/DOX@S/P-lip administration. **(C-F)** The liver and kidney function analysis of mice after drug administration. **(G)** The H&E staining of major organs (heart, liver, spleen, lung, and kidney) from C57BL/6 mice (Scale bars, 50 μm). **(H)** Pharmacokinetic profiles of AIE-MNPs/DOX@S/P-lip **(I)** Representative H&E staining of main organs from untreated and AIE-MNPs/DOX@S/P-lip treated mice at one month post-treatment; no evident pathological injury was observed in tested tissues (Scale bar 100 μm). Data are presented as mean±standard deviation. ns: no significance, *P < 0.05, **P < 0.01, ***P < 0.001, ****P < 0.0001.

**Figure S10. Proportion of cells undergoing total apoptosis or late apoptosis after various treatments. (A)** Proportion of RM1 cells undergoing total apoptosis. **(B–C)** Proportions of 22Rv1 cells undergoing total apoptosis and late apoptosis. **(D)** Proportion of PC-3 cells undergoing late apoptosis. (G1: liposomes; G2: DOX@lip; G3: AIE-MNPs/DOX@lip; G4: AIE-MNPs/DOX@S-lip; G5: AIE-MNPs/DOX@P-lip; G6: AIE-MNPs/DOX@S/P-lip). Data are presented as mean±standard deviation. ns: no significance, *P < 0.05, **P < 0.01, ***P < 0.001, ****P < 0.0001.

**Figure S11. *In vivo* targeting ability of AIE-MNPs/DOX@S/P-lip in C57BL/6 mice bearing RM1-STEAP1-PSMA tumor. (A)** Representative *in vivo* fluorescence images of tumor-bearing C57BL/6 mice at different time points (3, 6, 12, 24, and 48 h) after different treatment. (G1: liposomes; G2: AIE-MNPs/DOX@lip; G3: AIE-MNPs/DOX@S-lip; G4: AIE-MNPs/DOX@P-lip; G5: AIE-MNPs/DOX@S/P-lip; G6: AIE-MNPs/DOX@S/P-lip + external magnetic field). **(B)** Tumor-to-background ratios at the indicated time points. **(C–E)** *Ex vivo* fluorescence imaging **(C)** and quantitative analysis **(D, E)** of major organs at 48 h.

**Figure S12. *In vitro* investigation of the photothermal and magnetothermal effects of AIE-MNPs/DOX@S/P-lip.** (A) Temperature change of cell culture medium before and after 808 nm NIR laser irradiation for 5 min. (B) Temperature change of cell culture medium before and after 10 min application of an alternating magnetic field. (C) CCK-8 assay evaluating the growth inhibition of PCa cells by photothermal and magnetothermal effects of AIE-MNPs/DOX@S/P-lip (G1: PBS; G2: PBS + laser; G3: PBS + alternating magnetic field; G4: AIE-MNPs/DOX@S/P-lip; G5: AIE-MNPs/DOX@S/P-lip + laser; G6: AIE-MNPs/DOX@S/P-lip + alternating magnetic field; G7: AIE-MNPs/DOX@S/P-lip + laser + alternating magnetic field). (D–F) Flow cytometric analysis of apoptosis showing the cytotoxic effects and corresponding quantitative analysis of photothermal and magnetothermal effects of AIE-MNPs/DOX@S/P-lip on PC-3 (D), RM1 (E) and 22Rv1 (F) cells (G1: PBS; G2: PBS + laser; G3: PBS + alternating magnetic field; G4: AIE-MNPs/DOX@S/P-lip; G5: AIE-MNPs/DOX@S/P-lip + laser; G6: AIE-MNPs/DOX@S/P-lip + laser + alternating magnetic field). (G–I) Intracellular ROS levels in 22Rv1 (G), PC-3 (H), and RM1 (I) cells after various treatments (G1: PBS; G2: liposomes; G3: DOX; G4: DOX@lip; G5: AIE-MNPs/DOX@lip; G6: AIE-MNPs/DOX@S-lip; G7: AIE-MNPs/DOX@P-lip; G8: AIE-MNPs/DOX@S/P-lip; G9: AIE-MNPs/DOX@S/P-lip + laser). Each experiment was performed with three independent biological replicates for each group. Data are presented as mean ± standard deviation. ns: no significance, ***P < 0.001, ****P < 0.0001.

**Figure S13. Biosafety assessment of AIE-MNPs/DOX@S/P-lip administration in BALB/c nude mice. (A)** The body weight of mouse after drug administration (n = 5). **(B-E)** The liver and kidney function analysis of mice after drug administration.

**Figure S14. The H&E staining of major organs (heart, liver, spleen, lung and kidney) (G1: PBS; G2: liposomes; G3: DOX@lip; G4: AIE-MNPs/DOX@lip; G5: AIE-MNPs/DOX@S-lip; G6: AIE-MNPs/DOX@P-lip; G7: AIE-MNPs/DOX@S/P-lip; G8: PBS + laser; G9: AIE-MNPs/DOX@S/P-lip + laser). Scale bar: 20 μm.**

**Figure S15. *In vivo* safety in C57BL/6 mice. (A-D)** The liver and kidney function analysis of mice after drug administration. **(E)** The H&E staining of major organs (heart, liver, spleen, lung and kidney) (Scale bars, 50 μm).

**Figure S16. Flow-cytometry gating strategies. (A)** Gating strategy for DCs. **(B)** Gating strategy for macrophages. **(C)** Gating strategy for CD8⁺ T cells.

**Figure S17. *In Vivo* immunostimulatory effects of AIE-MNPs/DOX@S/P-lip (A-F)** Representative flow cytometry images and quantitative analysis of the proportion of CD80^+^DCs **(A)**, CD86^+^DCs **(B)**, iNOS^+^macrophages, Arg1^+^macrophages **(C)**, TNFα^+^macrophages **(D)**, CD69^+^ **(E)** and IFN-γ^+^ **(F)** CD8^+^T cells (G1: PBS; G2: DOX@lip; G3: AIE-MNPs/DOX@lip; G4: AIE-MNPs/DOX@S-lip; G5: AIE-MNPs/DOX@P-lip; G6: AIE-MNPs/DOX@S/P-lip). Data are presented as mean ± standard deviation. ns: no significance, *P < 0.05, **P < 0.01, ***P < 0.001, ****P < 0.0001.

**Figure S18. Therapeutic efficacy in right-sided tumors. (A) Ex** *vivo* images of right tumors after mouse sacrifice (n = 5) (G1: PBS; G7: AIE-MNPs/DOX@S/P-lip; G8: PBS + laser; G9: AIE-MNPs/DOX@S/P-lip + laser). **(B)** The tumor growth curves of right tumors (n = 5). **(C-D)** The comparison of tumor volume **(C)** and weight **(D)** of right tumors after mouse sacrifice (n = 5). **(E)** The body weight of mouse after drug administration (n = 5).

**Figure S19. In Vivo immunostimulatory effects of AIE-MNPs/DOX@S/P-lip combined with photothermal therapy.** (A-E) Representative flow cytometry images and quantitative analysis of the proportion of CD80+DCs (A), CD86+DCs (B), CD69+ (C), perforin+ (D) and TNFα+ (E) CD8+T cells (G1: PBS; G7: AIE-MNPs/DOX@S/P-lip; G8: PBS + laser; G9: AIE-MNPs/DOX@S/P-lip + laser). Data are presented as mean ± standard deviation. ns: no significance, *P < 0.05, **P < 0.01, ***P < 0.001, ****P < 0.0001.

**Figure S20. Gating strategy for memory T cells isolated from lymph nodes, spleens and PBMCs, and spleen size analysis.** Flow cytometry gating schemes of memory T cells from lymph nodes (A), spleens (B) and peripheral blood mononuclear cells (PBMCs, C), respectively. (D) The percentage of CD8⁺ T cells across groups G1 to G4. (E) Quantitative comparison of spleen weight among the four treatment cohorts (G1: blank; G2: PBS; G3: AIE-MNPs/DOX@S/P-lip; G4: AIE-MNPs/DOX@S/P-lip+laser).

**Supplementary Methods**

**Nanoscale Spatial Colocalization of Dual Antibodies via dSTORM**

Super-resolution dSTORM imaging was employed to characterize the nanoscale spatial organization of dual antibodies on the nanoparticle surface. Briefly, Cy5-labeled anti-STEAP1 and Cy3-labeled anti-PSMA functionalized nanoparticles were immobilized and imaged in TIRF mode using a Zeiss Elyra 7 system. 20,000 frames were captured per channel in a specialized switching buffer to induce stochastic fluorophore blinking. Image reconstruction and drift correction were performed using Zeiss Zen software, achieving a lateral resolution of ~20 nm. The resulting images and colocalization analysis confirm the successful dual-conjugation and uniform spatial distribution of STEAP1 and PSMA at the single-nanoparticle level.

**In vivo Biosafety Evaluation**

Systemic toxicity was evaluated in healthy C57BL/6 and BALB/c nude mice (n = 5 per group). Mice were administered AIE-MNPs/DOX@S/P-lip via intravenous injection at therapeutic doses (2, 3, and 5 mg/kg) or saline control once every three days for three cycles. Body weight was monitored. At 48 h after the final injection, mice were sacrificed and blood was obtained via cardiac puncture. Serum was isolated by centrifugation at 4°C, 3,000 ×g for 15 min and analyzed for liver function markers (alanine transaminase [ALT], alkaline phosphatase [ALP], and aspartate transaminase [AST]) and renal function markers (creatinine [CREA] and blood urea nitrogen [BUN]) with a Rayto Chemray 800 fully automated biochemical analyzer (China). Major organs were harvested. Tissues were fixed in 4% PFA, paraffin-embedded, cut into 5-μm sections, and H&E-stained. A blinded pathologist evaluated histopathology by light microscopy. For long-term safety evaluation, an additional cohort of mice received AIE-MNPs/DOX@S/P-lip at the highest therapeutic dose (5 mg/kg) via intravenous injection once every three days. One month after the final administration, mice were sacrificed, and major organs were harvested, fixed in 4% PFA, paraffin-embedded, sectioned at 5 μm, and stained with hematoxylin and eosin (H&E).

**Hemolysis Assay**

Hemocompatibility was assessed using mouse red blood cells (RBCs). Whole blood was collected into anticoagulant tubes, and RBCs were separated through centrifugation at 4°C (3000 rpm, 15 min). The erythrocyte pellet underwent 2 washing cycles with sterile 0.9% saline and resuspended to a 2% (v/v) hematocrit. Aliquots (1 mL) of RBC suspension were incubated for 4 h at 37°C with either 0.9% saline (negative control), deionized water (positive control), or AIE-MNPs/DOX@S/P-lip solutions at concentrations of 0.05, 0.1, 0.15, and 0.2 mg/mL. After incubation, samples were centrifuged again at 4°C, 3,000 rpm for 15 min, after which 0.1mL of the clarified supernatant was pipetted from each tube into a 96-well microplate. Hemolysis was determined by spectrophotometric analysis at 542 nm on a microplate reader. The percentage of hemolysis was calculated as: Hemolysis (%) = [(sample’s Abs – Abs of saline) / (Abs of H₂O – Abs of saline)] × 100%.

**Cell Viability Assay (CCK-8)**

The anti-proliferative activity of different treatments against PCa cells was evaluated using the CCK-8 assay (Abclonal, China). Briefly, PCa cells were seeded in 96-well plates at a density of 8 × 10³ cells per well and cultured for 12 h for adherence. Cells were then incubated with different formulations at predetermined concentrations for the designed duration. After treatment, the culture medium was removed, and cells were rinsed with PBS. Subsequently, fresh serum-free medium containing 10% CCK-8 solution was added to each well, followed by incubation at 37 °C for 2 h. The absorbance at 450 nm was measured, and cell viability was calculated accordingly.

**Colony and Migration Assays**

Prior to functional experiments, PCa cells were pretreated with serially diluted AIE-MNPs/DOX@S/P-lip nanocomplexes. Briefly, 3 × 10⁵ cells were seeded in 6-well plates and incubated for 12 h to allow full adherence. Post-treatment cells were digested with trypsin-EDTA and re-seeded into 6-well plates at a density of 800 cells per well. The culture medium was refreshed regularly. After 14–21 days of incubation until macroscopic colonies formed, cells were fixed and stained with 1% crystal violet solution. Colony images were captured and quantified using ImageJ software. Cell migration was evaluated using 8.0 μm-pore transwell inserts. In total, 8 × 10⁴ treated cells suspended in serum-free medium were seeded in the upper chamber, while the lower chamber was filled with medium containing 20% fetal bovine serum. After 48 h of incubation, non-migrated cells on the upper membrane were wiped off. Migrated cells on the lower membrane were fixed, stained with 0.5% crystal violet, and counted in five random fields under microscopy.

**Intracellular ROS Assay**

ROS generation was assessed using a ROS Detection Kit (Beyotime, China) per manufacturer instructions and analyzed by flow cytometry. PCa cells were seeded in 6-well plates, treated as indicated for 24 hours. Cells were loaded with 3 µM DCFH-DA in RPMI-1640 for half an hour. Fluorescence was measured on a BeamCyte-1026 flow cytometer (China) and analyzed with FlowJo v10.8.1 (USA).

**Apoptosis Assay**

Annexin V/7-AAD kit (KeyGEN, China) was used and cell apoptosis was detected by flow cytometry. PCa cells were seeded and treated for 24 h, then harvested. After supernatant removal, cells were trypsinized, washed, and stained per manufacturer instructions. Apoptotic cells were quantified on a BeamCyte-1026 flow cytometer (China) and analyzed with FlowJo v10.8.1 (USA).

**Table S1. Quantification of chemical elements by X-ray photoelectron spectroscopy**

| **Element** | **Atomic conc. [%]** | **Error [%]** | **Mass conc. [%]** | **Error [%]** |
| --- | --- | --- | --- | --- |
| Fe 2p | 31.59 | 0.41 | 63.48 | 0.42 |
| O 1s | 48.12 | 0.49 | 27.71 | 0.31 |
| N 1s | 0.55 | 0.40 | 0.28 | 0.20 |
| C 1s | 19.74 | 0.57 | 8.53 | 0.28 |
| S 2p | 0.08 | 0.05 | 0.21 | 0.12 |
| P 2p | 0.50 | 0.06 | 1.22 | 0.15 |

**Table S2. List of antibodies and reagents used in this study.**

| ***Antibodies*** | ***Company name*** | ***Identifier*** |
| --- | --- | --- |
| PE anti-mouse Arginase 1 Antibody | BioLegend | Cat#: 165803 |
| PE/Dazzle™ 594 anti-mouse F4/80 Antibody | BioLegend | Cat#: 123145 |
| PE/Cyanine7 anti-mouse/human CD11b Antibody | BioLegend | Cat#: 101215 |
| APC anti-mouse CD206 (MMR) Antibody | BioLegend | Cat#: 141707 |
| Alexa Fluor® 700 anti-mouse CD86 Antibody | BioLegend | Cat#: 159213 |
| APC/Cyanine7 anti-mouse CD45 Antibody | BioLegend | Cat#: 103115 |
| Brilliant Violet 421™ anti-mouse TNF-α Antibody | BioLegend | Cat#: 506327 |
| iNOS Antibody, anti-mouse, REAfinity™ | Miltenyi Biotech | Cat#: 130-116-357 |
| Zombie Aqua™ Fixable Viability Kit | BioLegend | Cat#: 423102 |
| FITC anti-mouse CD8a Antibody | BioLegend | Cat#: 162314 |
| PE/Dazzle™ 594 anti-mouse CD3 Antibody | BioLegend | Cat#: 100246 |
| Granzyme B Antibody, anti-human/mouse/rat, REAfinity™ | Miltenyi Biotech | Cat#: 130-120-703 |
| PE-CF594 Hamster Anti-Mouse CD3e | BD Horizon™ | Cat#: 562286 |
| PE anti-mouse Perforin Antibody | BioLegend | Cat#: 154305 |
| PerCP/Cyanine5.5 anti-mouse CD80 Antibody | BioLegend | Cat#: 104722 |
| FITC anti-mouse CD11c Antibody | BioLegend | Cat#: 117306 |
| Brilliant Violet 650™ anti-mouse/human CD44 Antibody | BioLegend | Cat#: 103049 |
| Brilliant Violet 785™ anti-mouse CD62L Antibody | BioLegend | Cat#: 104440 |
| Brilliant Violet 605™ anti-mouse I-A/I-E Antibody | BioLegend | Cat#: 107639 |
| anti-mouse CD8 Antibody | ABCAM | Cat#: ab217344 |
| anti-mouse CD68 Antibody | CST | Cat#: 97778T |
| anti-mouse CD206 Antibody | CST | Cat#: 24595T |
| anti-mouse CD11c Antibody | CST | Cat#: 97585T |
| anti-mouse GZMB Antibody | ABCAM | Cat#: ab255598 |
| anti-mouse CD86 Antibody | CST | Cat#: 19589T |
| HRP Goat anti-rabbit Antibody | Shanghai Ruchuang Bio | Cat#: RCA054 |
| Phospho-MLKL-S441 Rabbit pAb | ABClonal | Cat#: AP1174 |
| MLKL Rabbit mAb | ABClonal | Cat#: A26436 |
| RIPK1/RIP Rabbit mAb | ABClonal | Cat#: A19580 |
| Bax Rabbit mAb | ABClonal | Cat#: A19684 |
| Phospho-RIPK1-S166 Rabbit mAb | ABClonal | Cat#: AP1448 |
| Calreticulin Rabbit mAb | ABClonal | Cat#: A20986 |
| HMGB1 Rabbit mAb | ABClonal | Cat#: A19529 |
| CDKN1A/p21 Rabbit mAb | ABClonal | Cat#: A19094 |
| SQSTM1/p62 Rabbit mAb | ABClonal | Cat#: A19700 |
| LC3B Antibody | CST | Cat#: 2775 |
| Bcl-2 Rabbit mAb | ABClonal | Cat#: A19693 |
| Cleaved-Caspase 3 p12 Rabbit mAb | ABClonal | Cat#: A28349 |
| GAPDH Rabbit mAb | ABClonal | Cat#: A19056 |
| GPX4 Recombinant monoclonal antibody | Proteintech | Cat#: 82822-2-RR |
| ACSL4/FACL4 Recombinant monoclonal antibody | Proteintech | Cat#: 81196-1-RR |
| SLC7A11/xCT Polyclonal antibody | Proteintech | Cat#: 26864-1-AP |
